# Associations between flavan-3-ol intake and CVD risk in the Norfolk cohort of the European Prospective Investigation into Cancer (EPIC-Norfolk)

**DOI:** 10.1016/j.freeradbiomed.2015.03.005

**Published:** 2015-07

**Authors:** Anna Vogiatzoglou, Angela A. Mulligan, Amit Bhaniani, Marleen A.H. Lentjes, Alison McTaggart, Robert N. Luben, Christian Heiss, Malte Kelm, Marc W. Merx, Jeremy P.E. Spencer, Hagen Schroeter, Kay-Tee Khaw, Gunter G.C. Kuhnle

**Affiliations:** a Department of Food & Nutritional Sciences, University of Reading, UK; b Department of Public Health and Primary Care, University of Cambridge, UK; c Division of Cardiology, Pulmonology and Vascular Medicine, Medical Faculty, University Düsseldorf, Germany; d Mars, Inc., McLean, VA 22101, USA; e University of Cambridge, School of Clinical Medicine, Clinical Gerontology Unit, Cambridge, UK

**Keywords:** ECG, epicatechin-3′-gallate, EGC, epigallocatechin, EGCG, epigallocatechin-3′-gallate, GC, gallocatechin, CG, catechin-3′-gallate, EFSA, European Food Safety Authority, DP, degree of polymerization, 7DD, 7-day-diary, FFQ, food frequency questionnaire, CVD, cardiovascular disease, IHD, ischemic heart disease, EPIC, European Prospective Investigation into Cancer and Nutrition, Flavan-3-ols, Cardio-vascular diseases, Nutritional epidemiology, EPIC Norfolk

## Abstract

Dietary intervention studies suggest that flavan-3-ol intake can improve vascular function and reduce the risk of cardiovascular diseases (CVD). However, results from prospective studies failed to show a consistent beneficial effect. Associations between flavan-3-ol intake and CVD risk in the Norfolk arm of the *European Prospective Investigation into Cancer and Nutrition* (EPIC-Norfolk) were investigated. Data were available from 24,885 (11,252 men; 13,633 women) participants, recruited between 1993 and 1997 into the EPIC-Norfolk study. Flavan-3-ol intake was assessed using 7-day food diaries and the *FLAVIOLA Flavanol Food Composition database*. Missing data for plasma cholesterol and vitamin C were imputed using multiple imputation. Associations between flavan-3-ol intake and blood pressure at baseline were determined using linear regression models. Associations with CVD risk were estimated using Cox regression analyses. Median intake of total flavan-3-ols was 1034 mg/d (range: 0–8531 mg/d) for men and 970 mg/d (0–6695 mg/d) for women, median intake of flavan-3-ol monomers was 233 mg/d (0–3248 mg/d) for men and 217 (0–2712 mg/d) for women. There were no consistent associations between flavan-3-ol monomer intake and baseline systolic and diastolic blood pressure (BP). After 286,147 person-years of follow-up, there were 8463 cardiovascular events and 1987 CVD related deaths; no consistent association between flavan-3-ol intake and CVD risk (HR 0.93, 95% CI: 0.87; 1.00; Q1 vs Q5) or mortality was observed (HR 0.93, 95% CI: 0.84; 1.04). Flavan-3-ol intake in EPIC-Norfolk is not sufficient to achieve a statistically significant reduction in CVD risk.

## Highlights

• Median intake of total flavan-3-ol monomers in EPIC Norfolk was 233 mg/d for men and 217 mg/d for women.
• There were no consistent associations between flavan-3-ol monomer intake and baseline systolic and diastolic blood pressure in EPIC Norfolk.
• There were no consistent association between flavan-3-ol intake and CVD risk in EPIC Norfolk.
• Flavan-3-ol intake in EPIC-Norfolk is not sufficient to achieve a statistically significant reduction in CVD risk.

## Graphical abstract

**Figure:**
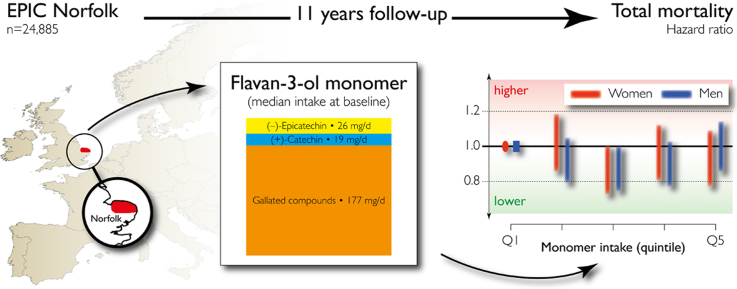

## Introduction

The medicinal use of flavan-3-ols and proanthocyanidins has a long documented history [1] and as early as 1590 in the *Florentine Codex* [2], cacao beans, a major source of flavanols, were recommended to treat heart disease [3]. In recent decades, there has been an increasing interest in the physiological effects of flavan-3-ols on vascular function. While their vasculoprotective properties were at first attributed to their activity as antioxidants *in vitro* [4], recent research has shown that this is unlikely [5] and that the modulation of arterial function of flavan-3-ols is based on a nitric oxide-dependent mechanism [6–8].

Dietary intervention studies with different sources of flavan-3-ols, such as cocoa flavanols [9–11] or grape seed extracts [12], have shown a potentially beneficial effect on vascular function. However, data from observational studies are more ambiguous. Data from anthropological research in a population with high habitual intake of cocoa, the Kuna of Panama, suggest that flavan-3-ol intake can improve vascular function [13], but results from the *Nurses Health Studies* and *Health Professionals Follow-up Study* [14–16]*,* the *Iowa Women׳s Health Study* [17], the *Cancer Prevention Study* [18], and the *Zutphen Elderly Study* [19] have not shown any consistent associations between flavan-3-ol intake and CVD risk or risk factors such as blood pressure. Studies investigating associations between CVD risk and intake of flavan-3-ol-rich foods, in particular chocolate, did show beneficial effects [20–22], but the flavan-3-ol contribution from chocolate in the European population is very small [23] and it is likely that self-reported chocolate consumption acts mainly as a surrogate marker for a yet unidentified factor.

The results from intervention studies suggest that flavan-3-ols can reduce the risk for CVD. This is of great importance in the context of public health, as CVDs are responsible for approximately 30% of all deaths globally [24]. However, the ambiguity of results from observational studies raises several questions, in particular how the apparent contradiction with intervention studies can be explained and whether these data can be easily translated to the general public.

The primary objective of this study was to investigate associations between flavan-3-ol intake and CVD risk in the Norfolk arm of the *European Prospective Investigation into Cancer and Nutrition* (EPIC-Norfolk), and to compare these results with data from previous studies as well as dietary intervention studies in the context of primary prevention of CVD. The EPIC-Norfolk cohort is particularly well placed to investigate these associations, as it has the widest range and highest intake of flavan-3-ol intake among all EPIC cohorts [25] and a wider range of intake than cohorts investigated previously. Furthermore, we have assessed intake using 7-day-diaries (7DD) and a newly developed food composition database [23], which provide a more accurate estimate of dietary intake.

## Subjects and methods

### Study population

Between 1993 and 1997, approximately 30,000 men and women between 40 and 75 years were recruited for the *European Prospective Investigation into Cancer and Nutrition* (EPIC) Norfolk study from a general practitioners׳ database [26]. The 25,639 participants attended a health examination. Diet was assessed by 7DD. The first day of the diary was completed as a 24-h recall (24HDR) with a trained interviewer and the remainder completed during subsequent days. Diary data were entered using the in-house dietary assessment software DINER—Data into Nutrients for Epidemiological Research [27]; data were checked and calculated using DINERMO [28]. Health and lifestyle characteristics, including data on smoking, alcohol consumption, social class, family medical history, exercise, and reproductive history, were assessed by questionnaire. Height and weight measurements were collected following a standardized protocol as part of a health check conducted by trained research nurses. Physical activity, representing occupational and leisure activity, was assessed using a validated questionnaire [29]. Blood pressure was measured by using a noninvasive oscillometric blood pressure monitor (Acutorr; Datascope Medical, Huntingdon, UK) after the participant had been seated in a comfortable environment for 5 min. The arm was horizontal and supported at the level of the mid-sternum; the mean of two readings was used for analysis. Acutorr readings were checked against those from sphygmomanometers every 6 months. The study was approved by the Norwich District Health Authority Ethics Committee, and all participants gave signed informed consent.

**Fig. 1:**
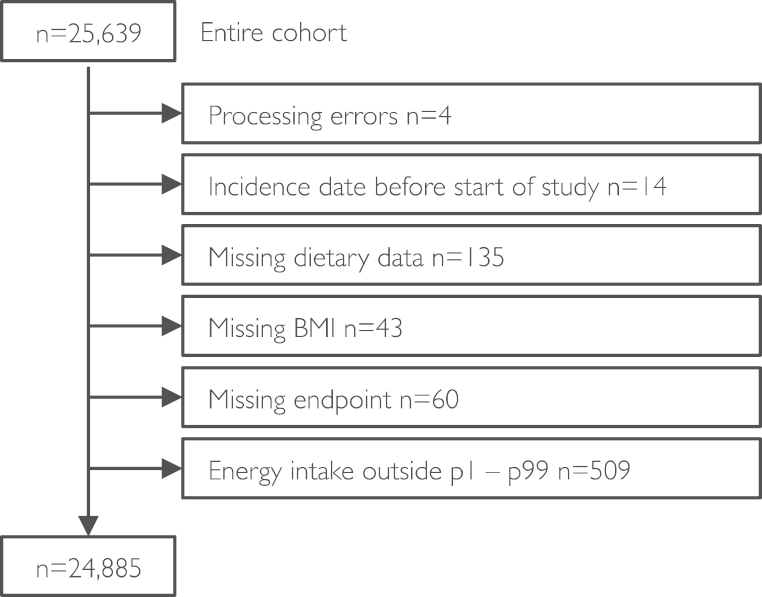
Study population and exclusion criteria.

Nonfasting blood samples were taken by vein puncture at baseline health check and stored in serum tubes. Serum levels of total cholesterol were measured on fresh samples with the RA 1000 autoanalyzer (Bayer Diagnostics, Basingstoke, UK). Plasma vitamin C was measured using a fluorometric assay as described previously [30]. Baseline data for 25,639 participants (11,607 men; 14,032 women) were available. Participants with data processing errors (4 men), incidence date before the start of the study (10 men; 4 women), and missing dietary data (73 men; 62 women) were excluded. Furthermore, participants with self-reported energy intake in the bottom (p1) or top (p99) sex-specific percentile (men, 4.6 MJ–15.3 MJ; women, 3.5 MJ–11.4 MJ) (230 men; 279 women), missing BMI (20 men; 23 women), and missing endpoint data (systolic blood pressure; 24 men; 36 women) were excluded, resulting in a sample size of 24,885 (11,252 men; 13,633 women; see also Fig. 1).

### Incident CVD event and mortality

The primary endpoint of this study was any first CVD event (defined as ICD 410–448 (ICD 9) or ICD I10–I79 (ICD 10), which includes coronary heart disease (410–414 (ICD 9) or I20–I25 (ICD 10)), stroke (430–438 (ICD 9) or I60–I69 (ICD 10)), and cardiac failure (428 (ICD 9) or I50 (ICD 10)) and other vascular causes) during follow-up. All participants were followed up for fatal and nonfatal CVD events, and the present study includes events until 31 March 2009, covering 11.1±3.7 years (mean±SD) of follow up. Cause-specific hospital admission was determined via ENCORE (East Norfolk Commission Record, the hospital admissions database kept by the East Norfolk Health Commission) [31] with the individuals׳ unique National Health Service number. All individuals were flagged by the UK Office of National Statistics (ONS) for death certification and trained nosologists coded death certificates according to the International Classification of Disease (ICD). Death certificates and ENCORE show high accuracy in correctly identifying incident disease, as previously shown in EPIC-Norfolk for incident stroke [31].

### Flavan-3-ol intake

We estimated flavan-3-ol intake by using the *FLAVIOLA Flavanol Food Composition database,* which was developed for the FLAVIOLA project [23]. The database contains food composition data for approximately 3000 food items and is based on the US Department of Agriculture database for the Flavonoid Content of Selected Foods [32] and the Proanthocyanidins (PA) Content of Selected Foods [33], expanded with values from Phenol-Explorer, a comprehensive database on the polyphenol content of foods [34]. These databases are the most up-to-date databases on flavonoids and polyphenols, and include information for 500, 205, and 456 food items for flavonoids [32], PA [33], and polyphenols [34], respectively. On the basis of these data, we determined the flavan-3-ol content of foods by substitution and calculations to reflect edible portions, food preparation methods, and recipe break down (see [35] for the methodology). Flavan-3-ol intake was determined for the following groups of flavan-3-ols (Table 1): flavan-3-ols monomers [(−)-epicatechin, (−)-epicatechin-3-gallate, (+)-catechin, (−)-epigallocatechin, (−)-epigallocatechin-3-gallate, (+)-gallocatechin, (+)-catechin-3-gallate], theaflavins (theaflavin, thearubigins, theaflavin-3,3′-digallate, theaflavin-3′-gallate, theaflavin-3-gallate), and PA (dimers, trimers, 4–6 mers, 7–10 mers, >10 mers).

### Data analysis

#### Missing data

Supplemental Table 1 shows a summary of missing data. For categorical data, an additional value of “missing” was created. For continuous variables (cholesterol and plasma vitamin C), missing values were assumed to be missing at random and were imputed using multiple imputation (*n*=5) with univariate regression models.

#### Descriptive statistics

Descriptive characteristics of the study population were summarized using mean (plus or minus standard deviation) for normally distributed continuous variables, frequency and percentages for categorical variables, and geometric means (standard deviation) for nonnormally distributed continuous variables.

#### Association between flavan-3-ol intake and blood pressure

Associations between flavan-3-ol intake and systolic blood pressure were investigated using linear regression models; dietary intake data were divided into sex-specific quintiles. Log-transformed values were used for the dependent variable (systolic blood pressure), as well as for BMI (body weight/height^2^ in kg/m^2^) and the intake of energy, sodium, potassium, fat, and saturated fat. In initial analyses (model 1), intake was adjusted by age, BMI (kg/m^2^) and energy intake (kJ/d (day)). A parsimonious model was developed using a stepwise selection approach (only covariables with a *P* value<0.2 were included in the model) using the following covariables: BMI (kg/m^2^), energy intake (kJ/d), plasma vitamin C (µmol/L), fiber, total fat and saturated fat consumptions (g/day), and alcohol consumption (0 g/d, <15 g/d, ≥15 g/d) and the following categorical variables—physical activity (inactive, moderately inactive, moderately active, active), smoking status (former, current, never), social class (professional, managerial, skilled (manual/nonmanual), semiskilled, unskilled), marital status (single, married, widowed, divorced, separated), education (none, O-level, A-level, degree), self-reported history of stroke, myocardial infarction and diabetes at baseline, family history of myocardial infarction, use of antihypertensive and lipid lowering drugs; additionally in women hormone replacement therapy (current, former, never) and menopausal status (pre, peri, and post). The parsimonious model included age, BMI, energy intake, plasma vitamin C, dietary intake of fiber, fat, saturated fat, and alcohol and the following categorical variables: physical activity, smoking status, marital status, education, use of antihypertensive drugs, and self-reported history of stroke, myocardial infarction, and diabetes at baseline, family history of myocardial infarction and—for women—menopausal status and hormone replacement therapy. Tests for linear trend were conducted by including the median value of each quintile in the model.

#### Association between flavan-3-ol intake and CVD risk

Associations between quintiles of flavan-3-ol intake and CVD risk and mortality were analyzed using a Cox regression analysis to investigate hazard ratios and 95% confidence intervals. Log-transformed values were used for systolic blood pressure, BMI, and the intakes of energy, sodium, potassium, fat, and saturated fat. Associations were first analyzed adjusting for BMI and energy intake only. A parsimonious model was then developed as described above, and it included the following variables: BMI, energy intake, systolic blood pressure, plasma cholesterol, plasma vitamin C, intake of fiber, potassium, sodium, fat, saturated fat, and alcohol, and the following categorical variables—physical activity, smoking status, education, self-reported history of stroke, myocardial infarction and diabetes at baseline, family history of myocardial infarction, use of antihypertensive and lipid lowering drugs; additionally in women use of hormone replacement therapy. Tests for linear trend were conducted by including the median value of each quintile in the model.

#### Sensitivity analyses

In post hoc sensitivity analyses, we investigated associations in participants healthy at baseline (no self-reported use of antihypertensive drugs, no self-reported incidence of diabetes mellitus, myocardial infarction, or stroke), those at high risk of CVD (10-year CVD risk 20% higher than normal based on Framingham risk score [36]) and those with at least 3 days of dietary data.

#### Strobe checklist

The STROBE checklist for cohort studies [37] has been completed for this study.

### Statistical analyses

All analyses were conducted with Stata (version 11 for Mac OS X; Stata Corp, College Station, TX, USA).

## Results

### Study population and flavan-3-ol monomer intake

Data were available for 11,252 men and 13,633 women, 8176 of which were postmenopausal. Baseline characteristics of the cohort and dietary information by sex-specific quintile of flavan-3-ol monomer intake are shown in Supplemental Table 2. Mean age of the participants at baseline was 59 years (range: 39–79 years) for men and 58 years (39–78 years) for women. Median intake of total flavan-3-ols was 1034 mg/d (0–8531 mg/d) for men and 970 mg/d (0–6695 mg/d) for women; median intake of flavan-3-ol monomers was 233 mg/d (0–3248 mg/d) for men and 217 (0–2712 mg/d) for women (see Table 2 for more details). While there were significant differences in many baseline characteristics between sex-specific quintiles of flavan-3-ol monomer intake, these differences did not follow a consistent trend.

**Fig. 2:**
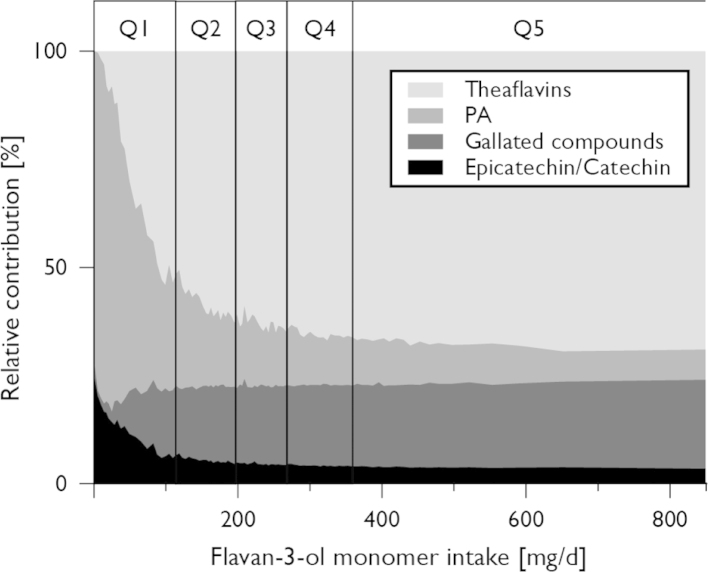
Relative composition of total dietary flavan-3-ols by flavan-3-ol monomer intake in male participants of EPIC-Norfolk (results for women and postmenopausal women are similar). (−)-Epicatechin and (+)-catechin were the main contributors of total monomer intake in the bottom quintile, while gallated compounds were the main contributor to monomer intake in the top four quintiles. For concomitantly consumed nonmonomeric flavanols, similar differences were observed. In the bottom quintile, proanthocyanidins were the dominant class of compounds, while in the other quintiles, theaflavins were more important.

The relative composition of dietary flavan-3-ol intake was consistent in the top four quintiles of flavan-3-ol intake (Q2 to Q5), with gallated compounds being the main contributor to total intake of monomers (Fig. 2; the same pattern was observed for women and postmenopausal women) and theaflavins the main type of oligomeric flavan-3-ols. However, in participants with low flavan-3-ol intake (Q1), (−)-epicatechin and (+)-catechin were the main sources of flavan-3-ol monomers and proanthocyanidins the main type of oligomeric flavan-3-ols consumed. It was also noticeable that the relative composition of dietary flavan-3-ols was more variable in the bottom quintile.

### Associations among flavan-3-ol intake, CVD risk, and mortality

In women, flavan-3-ol intake was not associated with CVD risk or mortality and these results were consistent when using different models and also in sensitivity analyses (Table 3). In men, flavan-3-ol intake was not associated with mortality and the risk of stroke. However, the association between flavan-3-ol intake and both IHD risk and total CVD risk was more complex and followed a J-shaped curve: low and medium intake (Q2 and Q3) were both associated with a reduced risk. The risk estimates for quintiles two to five of flavan-3-ol intake were not significantly different and there was no linear trend.

**Fig. 3:**
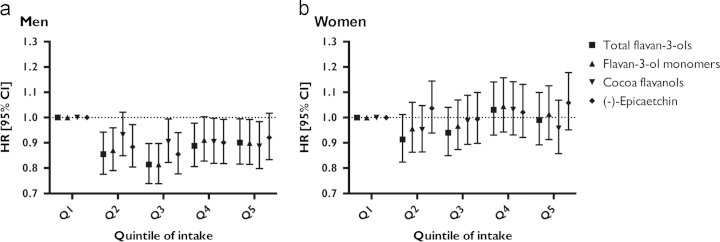
Association between flavan-3-ol intake and risk of CVD in 11,253 men (a) and 13634 women (b) of EPIC-Norfolk. This figure shows HR (95% CI) estimated using the parsimonious model and quintiles of intake of each respective flavan-3-ol group.

Post hoc sensitivity analyses with healthy individuals, individuals with a raised 10-year CVD risk, and those with at least 3 days of diary data showed no materially different associations. In sensitivity analyses, we investigated the association between specific groups of flavan-3-ols and CVD risk: no material differences were observed between different types of flavan-3-ols (Fig. 3).

### Associations between flavan-3-ol intake and blood pressure

Supplemental Table 4 shows the results of regression analyses between flavan-3-ol intake and systolic and diastolic blood pressure (log-transformed) at baseline. There were no consistent significant association between flavan-3-ol intake and baseline blood pressure.

## Discussion

The EPIC-Norfolk cohort is well positioned for a detailed investigation of the association between flavan-3-ol intake and CVD risk, in particular because of the prospective design of the study, the wide range of flavan-3-ol intake, and the detailed dietary information available from 7-day diet diaries. In combination with the newly developed *FLAVIOLA Flavanol Food Composition Database*, this provides the most accurate estimate of flavan-3-ol consumption in a prospective cohort currently available. There are some limitations, in particular the known limitations of self-reported dietary assessment and the accuracy of phytochemical intake based on food composition tables. We have also conducted multiple comparisons without adjustment, which should be considered when interpreting results.

**Fig. 4:**
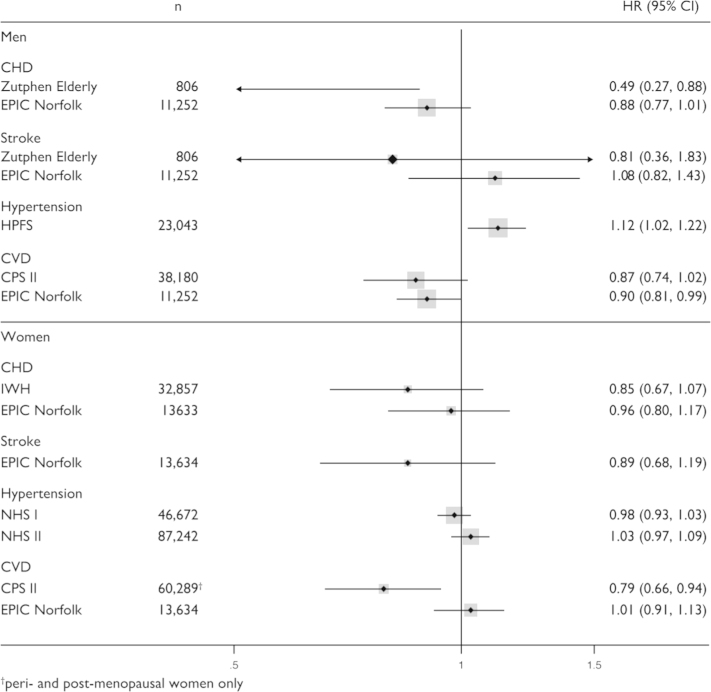
Association between flavan-3-ol intake and incidence of CVD in observational epidemiological studies conducted previously. Data show the estimated HR (95% CI) comparing the bottom and top quintile of intake. Studies included: Zutphen Elderly Study [20]; EPIC-Norfolk (this study); HPFS: Health-Professional Follow-Up Study [15]; CPS: Cancer Prevention Study II [18]; IWH: Iowa Women׳s Health Study [17]; NHS I and II: Nurses Health Study [15]. Data for IHD and CHD were combined.

The estimated flavan-3-ol monomer intake in this study was higher than in comparable studies conducted previously (Supplemental Table 4). Despite the wider range of intake, we have not found any consistent association between flavan-3-ol monomer intake and systolic blood pressure at baseline or risk of CVD, and our data are corroborated by results from previous studies [15,17,18,20,38] (Fig. 4 and Supplemental Table 4).

It was noticeable that the association between flavan-3-ol intake and CVD risk followed a J-shaped curve with the lowest relative risk in quintile 2, and this pattern has also been observed in previous studies [15,17,18]. Our data show differences in the relative composition of flavan-3-ols consumed between those in the bottom quintile of intake and other participants (Fig. 2). While data on flavan-3-ol composition in the bottom quintile should be interpreted carefully, because of the error associated with the assessment of such small amounts, it is likely that this difference in relative flavan-3-ol composition is an explanation for the J-shaped associations we observed.

The absence of consistent statistically significant associations between flavan-3-ol intake and CVD risk is noteworthy in light of data from dietary intervention studies, which suggest vasculoprotective effects of dietary flavan-3-ols. Indeed, several meta-analyses have shown a significant reduction in systolic blood pressure (3 to 5 mm Hg) for interventions with cocoa flavanols [10,11] and smaller reductions with other flavan-3-ol-rich foods such as tea [39] or grape seed extract [40]. A reduction of systolic blood pressure by 3 to 5 mm Hg is comparable to the effect that could be achieved by a modest reduction in salt intake [41]. Projections based on the DASH diet and data from NHANES estimate that such a reduction in blood pressure could translate into approximately 3–4% fewer CHD events over 10 years [42]. However, a direct comparison of results from intervention studies with data from this study is limited because observational epidemiological studies investigate by their very nature chronic effects, whereas most intervention studies focused on acute and subacute effects (see Supplemental Table 5 for a summary of dietary intervention studies), and there is a paucity of data on chronic effects. There is also considerable ambiguity regarding the type of flavan-3-ols used. While in observational studies all but one, the Iowa Women׳s Health Study [17], used flavan-3-ol monomers as defined here (Table 1); this is different for dietary intervention studies. In some intervention studies, flavan-3-ols are not characterized by compound-specific analyses, but only by their source. However, differences in growing conditions, harvesting, processing, and storage can result in a large variation of flavan-3-ol content in foods [43–46]. For these reasons, the actual flavan-3-ol contents, and their relative composition, are often not known. This is particularly important because so far only the intake of one member of the cocoa flavanol group, (−)-epicatechin, has directly and causally been linked to the modulation of vascular function [7,8]. The absorption of oligomeric proanthocyanidins, important constituents of cocoa flavanols and grape seed extracts, is negligible and they do not contribute to the pool of flavan-3-ol metabolites [47].

**Fig. 5:**
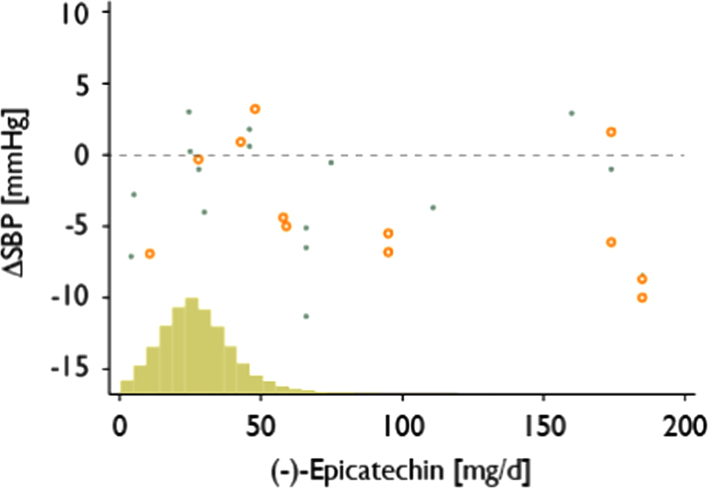
Dietary intervention studies investigating the effect of dietary flavan-3-ols on systolic blood pressure (see Supplemental Table 4 for details) based on (−)-epicatechin intake. Circles indicate studies, which were blinded, conducted for at least 28 days and provided information on (−)-epicatechin based on analyses. All other studies with a duration of 14 days or more are indicated by an X. (−)-Epicatechin intake was estimated as 10% of cocoa flavanol intake for studies where no analytical data were available. The histogram shows the distribution of intake in EPIC-Norfolk. Error bars were omitted to improve clarity.

**Fig. 6:**
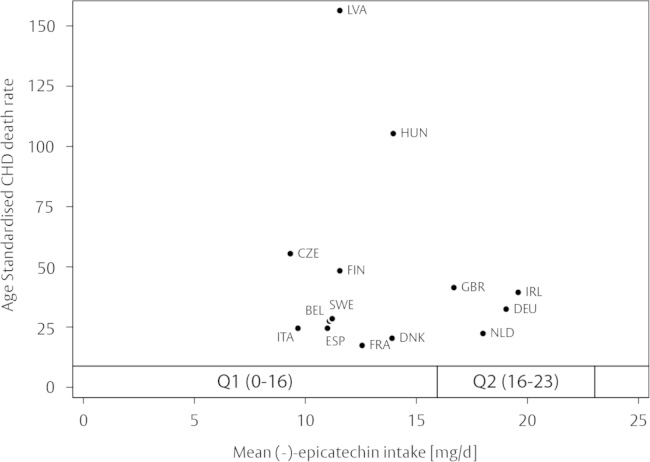
Mean (−)-epicatechin intake [23] and age-standardized CHD mortality (per 100,000, data for 2005) [54] in European countries. (−)-epicatechin intake in EPIC-Norfolk is shown for the relevant quintiles.

Using food composition tables, we have estimated the (−)-epicatechin intake in dietary intervention studies, assuming that cocoa flavanols consist of approximately 10% (−)-epicatechin, 7% (+)-catechin, and 83% proanthocyanidins (DP 2–10). A comparison of intakes used in intervention studies and the intake of the population in EPIC-Norfolk (Fig. 5) shows clearly that intakes in most studies were considerably higher, and it is likely that (−)-epicatechin consumption in EPIC-Norfolk, and other cohorts, is too low to have an effect on overall CVD risk. Indeed, a recent meta-analysis has suggested that an effect on systolic blood pressure can only be observed with intakes in excess of 50 mg/d [9] of (−)-epicatechin, but more than 90% of the EPIC-Norfolk population do not consume this much, even though the Norfolk cohort of EPIC has the highest (−)-epicatechin intake of all EPIC cohorts [25] and participants consume considerably more than the general European public (mean intake in the EU: 13.5 mg/d) [23]. A comparison of mean (−)-epicatechin in different European countries with age-adjusted CHD mortality does not suggest a significant association (Fig. 6). These data suggest that (−)-epicatechin intake in the general public is too low to achieve an overall reduction of cardiovascular disease risk.

In 2012, EFSA approved a health claim for cocoa flavanols, based on an unspecified degree of increase in flow-mediated dilation (FMD) [48] after cocoa flavanol intake. The scientific opinion underlying this claim states that 200 mg/d of cocoa flavanols can achieve improved vascular function. Independent of these findings, studies have shown that a FMD increase of 1 to 1.5% could translate in a reduction of CVD events by 10% [49], and such a reduction has been achieved in dietary intervention studies with cocoa flavanols [9]. In EPIC-Norfolk, approximately 25% of participants consumed 200 mg/d or more of cocoa flavanols. However, there was no significant difference in CVD risk between those who met the 200 mg/d recommendation and those who did not (HR 0.97; 95% CI: 0.89; 1.04 for men; HR 0.96; 95% CI: 0.89; 1.05 for women). When basing the intake recommendation on the known bioactive constituent of cocoa flavanols, (−)-epicatechin, more than 70% of participants met or exceed the recommendations of 20 mg/d. We observed no statistically significant association between intake above 20 mg/d and CVD risk in women (HR 1.00; 95% CI: 0.94; 1.08). However, this analysis is more complex in men as all participants with an (−)-epicatechin intake of less than 20 mg/d are in the bottom quintile of (−)-epicatechin and flavan-3-ol monomer intake. The small, but significant, association between CVD risk and (−)-epicatechin intake found for men (HR 0.92; 95% CI: 0.86; 0.99) could therefore also be explained by differences in dietary patterns as discussed above and needs to be confirmed in further studies.

Many dietary recommendations focus primarily on the prevention of deficiency diseases [1], but there is an increasing interest in also focusing on *bioactives* in the context of disease risk reduction and primary prevention [50]. There is accumulating evidence for the vasculoprotective effect of dietary flavan-3-ols and the observed effect on vascular function suggests that an adequate intake could result in a 10% reduction of CVD risk in the general public. This has important implications in the context of primary prevention of CVD and evidence-based dietary recommendations. So far, it is not yet clear what an adequate intake is: neither is it clear whether it should be based on individual compounds, such as (−)-epicatechin, or a group of compounds such as cocoa flavanols, nor is it clear which amount should be recommended. Nevertheless, the data presented here clearly show that the habitual intake in Europe or the United States is unlikely to have any significant effect on CVD risk on a population basis. Indeed, for such a reduction to occur, an intake considerably higher than the amount currently consumed by the general public is necessary.

Based on these data and previous investigations, it is essential that new methods are developed that enable further research to study more comprehensively the influences of age, sex, and dietary background. Specifically in the context of observational population-based studies, improved dietary assessment methods are crucial for a better understanding of associations between intake and CVD risk. Nutritional biomarkers can provide such methods, but there is currently no commonly accepted, validated biomarker of flavan-3-ol intake, which meets the recommendations of the 2010 IOM biomarker report [51]. The short half-life plasma in of structurally related flavan-3-ol metabolites [52] restricts their use to the assessment of acute intake. We have hypothesized that flavan-3-ol metabolites of the colonic microbiome, such as 5-(3,4-dihydroxyphenyl)-*γ*-valerolactone (*γ*-VL) [53], which have a considerably longer biological half-life and are present in higher concentrations [47], are more suitable as nutritional biomarkers of flavan-3-ol intake. In order to investigate these compounds on a large scale, we have developed a validated analytical method which can be automated for high-throughput analysis. We are currently applying it to a cohort of approximately 25,000 participants to assess colonic metabolites as nutritional biomarkers against the IOM criteria. Such a biomarker will then allow us a much more accurate estimate of intake.

Only a combination of data from observational and intervention studies will ultimately enable a comprehensive assessment of the impact of flavan-3-ol intake in the context of primary prevention, public health and evidence-based dietary recommendations.

## Figures and Tables

**Table 1:** Definition of different types of flavan-3-ols used in this study.

	Flavan-3-ols	Flavan-3-ol Monomer	Cocoa flavanols
(−)-Epicatechin	X	X	X
(+)-Catechin	X	X	X
Gallated forms^a^	X	X	X
Proanthocyanidins	X		X^b^
Thearubigins and Theaflavins^c^	X		

a Epicatechin-3′-gallate, epigallocatechin, epigallocatechin-3′-gallate, gallocatechin, catechin-3′-gallate.

b Includes only proanthocyanidins up to a degree of polymerization of 10.

c Theaflavin, thearubigins, theaflavin-3,3′-digallate, theaflavin-3′-gallate, theaflavin-3-gallate.

**Table 2:** Baseline characteristics (mean and SD) and baseline intake (median and range) of 11,252 men and 13,633 women in EPIC-Norfolk.

	Men	Women	Postmenopausal women
*n*	11252	13633	8176
Age [years]	59 (9)	58 (9)	64 (7)
BMI [kg/m^2^]	27 (3)	26 (4)	27 (4)
Cholesterol [mmol/L]	6.0 (1.1)	6.3 (1.2)	6.6 (1.2)
Systolic BP [mm Hg]	137.5 (17.7)	134.0 (18.9)	137.5 (17.7)
Diastolic BP [mm Hg]	84.4 (11.1)	81.0 (11.1)	82.8 (11.2)
Flavan-3-ol intake in mg/d			
Flavan-3-ols	1034 (0–8531)	970 (0–6695)	981 (0–6695)
Flavan-3-ol monomers	233 (0–3248)	217 (0–2712)	219 (0–2712)
Epicatechin	27 (0–225)	25 (0–198)	25 (0–198)
Catechin	20 (0–108)	18 (0–84)	17 (0–84)
Gallated compounds	183 (0–2936)	172 (0–2453)	176 (0–2453)
Proanthocyanidins^a^	138 (0–2082)	136 (0–1403)	134 (0–1359)
Theaflavins	644 (0–6095)	601 (0–4876)	612 (0–4876)
Cocoa flavanols^b^	154 (0–1561)	144 (0–1118)	142 (0–1060)

a With a degree of polymerization of two and above.

b (−)-Epicatechin, (+)-catechin, and proanthocyanidins with a degree of polymerization of 2 to 10.

**Table 3:** Association between flavan-3-ol intake and risk of CVD and mortality: Hazard ratio and 95% confidence interval a

		Q1	Q2	Q3	Q4	Q5	*p*_Trend_
Men (*n*=11,252)							
CVD Incidence (4403)	Person-Years (all men)	25291	24964	24980	24466	25001	
	Incidents (all men)	800	878	898	952	875	
	Model 1^b^	1	0.89 (0.81; 0.98)	0.84 (0.77; 0.93)	0.99 (0.90; 1.09)	0.97 (0.88; 1.07)	0.678
	Model 2^c^	1	0.87 (0.79; 0.96)	0.81 (0.74; 0.90)	0.91 (0.83; 1.00)	0.90 (0.81; 0.99)	0.176
	– healthy at baseline^d^ (*n*=8636)	1	0.86 (0.76; 0.97)	0.79 (0.70; 0.90)	0.98 (0.86; 1.10)	0.89 (0.78; 1.01)	0.427
	– high risk^e^ (*n*=8010)	1	0.85 (0.77; 0.95)	0.80 (0.72; 0.89)	0.87 (0.78; 0.97)	0.91 (0.82; 1.02)	0.270
	– at least 3 days diary (*n*=10,343)	1	0.85 (0.77; 0.94)	0.82 (0.75; 0.91)	0.91 (0.83; 1.01)	0.91 (0.82; 1.01)	0.329
IHD Incidence (2335)	Person-Years	27351	27293	27371	26872	27413	
	Incidents	425	456	459	531	464	
	Model 1^b^	1	0.89 (0.78; 1.02)	0.84 (0.74; 0.96)	1.06 (0.93; 1.20)	0.99 (0.87; 1.13)	0.309
	Model 2^c^	1	0.85 (0.74; 0.97)	0.81 (0.71; 0.93)	0.94 (0.83; 1.08)	0.88 (0.77; 1.01)	0.338
	– healthy at baseline^d^ (*n*=8636)	1	0.84 (0.70; 1.02)	0.75 (0.62; 0.91)	1.07 (0.89; 1.28)	0.97 (0.80; 1.17)	0.443
	– high risk^e^ (*n*=8010)	1	0.81 (0.70; 0.93)	0.80 (0.69; 0.92)	0.85 (0.74; 0.98)	0.86 (0.74; 1.00)	0.161
	– at least 3 days diary (*n*=10,343)	1	0.85 (0.74; 0.98)	0.85 (0.74; 0.98)	0.97 (0.84; 1.11)	0.94 (0.81; 1.08)	1.000
Stroke Incidence (5 9 5)	Person-Years	29575	29515	29613	29686	29614	
	Incidents	89	123	135	117	131	
	Model 1^b^	1	0.99 (0.75; 1.30)	0.99 (0.75; 1.29)	0.94 (0.71; 1.24)	1.18 (0.90; 1.54)	0.263
	Model 2^c^	1	0.99 (0.75; 1.31)	0.98 (0.75; 1.29)	0.88 (0.66; 1.17)	1.08 (0.82; 1.43)	0.727
	– healthy at baseline^d^ (n=8636)	1	1.13 (0.78; 1.64)	1.00 (0.68; 1.45)	1.04 (0.71; 1.52)	1.10 (0.75; 1.62)	0.765
	– high risk^e^ (*n*=8010)	1	0.96 (0.72; 1.30)	0.94 (0.70; 1.26)	0.89 (0.66; 1.20)	1.15 (0.85; 1.54)	0.388
	– at least 3 days diary (*n*=10,343)	1	0.87 (0.66; 1.15)	0.86 (0.66; 1.13)	0.79 (0.60; 1.05)	0.95 (0.71; 1.25)	0.670
CVD mortality (1154)	Person-Years	27854	27415	27550	27495	27533	
	Incidents	173	243	264	250	224	
	Model 1^b^	1	1.00 (0.80; 1.26)	0.96 (0.77; 1.21)	1.07 (0.85; 1.34)	1.07 (0.85; 1.35)	0.428
	Model 2^c^	1	0.98 (0.78; 1.23)	0.94 (0.75; 1.19)	0.94 (0.75; 1.18)	0.95 (0.74; 1.20)	0.607
	– healthy at baseline^d^ (*n*=8636)	1	1.15 (0.79; 1.68)	1.10 (0.76; 1.59)	1.44 (1.00; 2.07)	1.26 (0.86; 1.84)	0.123
	– high risk^e^ (*n*=8010)	1	1.04 (0.81; 1.32)	0.95 (0.74; 1.21)	0.96 (0.75; 1.23)	1.00 (0.78; 1.29)	0.852
	– at least 3 days diary (*n*=10,343)	1	0.91 (0.72; 1.15)	0.90 (0.72; 1.14)	0.93 (0.73; 1.17)	0.93 (0.73; 1.18)	0.677
All cause mortality (3244)	Person-Years	27854	27415	27550	27495	27533	
	Incidents	538	653	690	685	678	
	Model 1^b^	1	0.90 (0.79; 1.04)	0.85 (0.74; 0.97)	0.93 (0.81; 1.06)	1.03 (0.90; 1.18)	0.373
	Model 2^c^	1	0.91 (0.80; 1.05)	0.87 (0.76; 1.00)	0.90 (0.78; 1.03)	0.99 (0.86; 1.14)	0.946
	– healthy at baseline^d^ (*n*=8636)	1	1.00 (0.83; 1.21)	0.99 (0.82; 1.19)	1.07 (0.89; 1.28)	1.16 (0.97; 1.40)	0.065
	– high risk^e^ (*n*=8010)	1	0.92 (0.79; 1.06)	0.86 (0.74; 1.00)	0.87 (0.75; 1.01)	1.01 (0.86; 1.17)	0.894
	– at least 3 days diary (*n*=10,343)	1	0.88 (0.76; 1.01)	0.84 (0.73; 0.97)	0.91 (0.79; 1.04)	0.99 (0.86; 1.14)	0.709
Women (*n*=13,633)							
CVD Incidence (4060)	Person-Years	32794	32236	31938	31954	32524	
	Incidents	678	824	905	874	779	
	Model 1^b^	1	0.98 (0.89; 1.09)	1.01 (0.92; 1.12)	1.09 (0.98; 1.21)	1.11 (1.00; 1.23)	0.009
	Model 2^c^	1	0.96 (0.86; 1.06)	0.97 (0.87; 1.07)	1.04 (0.94; 1.16)	1.01 (0.91; 1.13)	0.364
	– healthy at baseline^d^ (*n*=10,795)	1	1.06 (0.92; 1.21)	0.97 (0.85; 1.11)	1.04 (0.91; 1.19)	1.12 (0.98; 1.28)	0.138
	– high risk^e^ (*n*=8507)	1	0.93 (0.83; 1.04)	0.95 (0.85; 1.06)	1.04 (0.93; 1.16)	0.96 (0.86; 1.08)	0.884
	– at least 3 days diary (*n*=12,815)	1	0.94 (0.85; 1.05)	0.96 (0.87; 1.06)	1.04 (0.93; 1.15)	1.01 (0.91; 1.13)	0.373
IHD incidence (1325)	Person-Years	35139	35167	35094	34897	35275	
	Incidents	207	269	306	313	230	
	Model 1^b^	1	1.01 (0.84; 1.22)	1.09 (0.91; 1.30)	1.25 (1.05; 1.50)	1.09 (0.91; 1.32)	0.076
	Model 2^c^	1	1.00 (0.83; 1.20)	1.06 (0.88; 1.27)	1.16 (0.97; 1.39)	0.96 (0.80; 1.17)	0.810
	– healthy at baseline^d^ (*n*=10,795)	1	1.15 (0.89; 1.49)	0.98 (0.76; 1.27)	1.20 (0.93; 1.54)	1.06 (0.81; 1.38)	0.648
	– high risk^e^ (*n*=8507)	1	1.01 (0.83; 1.23)	1.03 (0.85; 1.25)	1.20 (0.99; 1.45)	0.97 (0.78; 1.19)	0.748
	– at least 3 days diary (*n*=12,815)	1	1.04 (0.86; 1.25)	1.07 (0.89; 1.29)	1.20 (1.00; 1.45)	0.98 (0.80; 1.19)	0747
Stroke incidence (6 0 5)	Person-Years	35935	35994	36089	36022	36005	
	Incidents	96	132	140	129	108	
	Model 1^b^	1	0.96 (0.73; 1.25)	0.91 (0.70; 1.18)	0.95 (0.73; 1.25)	1.03 (0.78; 1.36)	0.806
	Model 2^c^	1	0.94 (0.72; 1.23)	0.86 (0.66; 1.12)	0.88 (0.67; 1.16)	0.89 (0.68; 1.19)	0.384
	– healthy at baseline^d^ (*n*=10,795)	1	1.00 (0.69; 1.44)	0.96 (0.67; 1.38)	1.00 (0.69; 1.44)	1.01 (0.69; 1.48)	0.958
	– high risk^e^ (*n*=8507)	1	0.93 (0.70; 1.24)	0.82 (0.62; 1.09)	0.83 (0.62; 1.11)	0.91 (0.68; 1.23)	0.393
	– at least 3 days diary (*n*=12,815)	1	0.91 (0.69; 1.19)	0.86 (0.66; 1.13)	0.87 (0.66; 1.15)	0.91 (0.68; 1.21)	0.502
CVD mortality (8 3 3)	Person-Years	3479	34597	34801	34652	35037	
	Incidents	138	173	188	193	141	
	Model 1^b^	1	0.95 (0.73; 1.25)	0.84 (0.64; 1.11)	0.98 (0.75; 1.28)	0.93 (0.70; 1.25)	0.734
	Model 2^c^	1	0.92 (0.70; 1.21)	0.78 (0.60; 1.03)	0.89 (0.67; 1.17)	0.81 (0.60; 1.09)	0.166
	– healthy at baseline^d^ (*n*=10,795)	1	1.02 (0.69; 1.51)	0.76 (0.51; 1.13)	0.76 (0.50; 1.15)	0.99 (0.65; 1.50)	0.566
	– high risk^e^ (*n*=8507)	1	0.95 (0.71; 1.26)	0.76 (0.57; 1.02)	0.88 (0.66; 1.17)	0.88 (0.64; 1.19)	0.322
	– at least 3 days diary (*n*=12,815)	1	0.97 (0.73; 1.28)	0.79 (0.60; 1.05)	0.90 (0.68; 1.20)	0.86 (0.64; 1.16)	0.280
All cause mortality (2657)	Person-Years	3479	34597	34801	34652	35037	
	Incidents	437	582	587	586	465	
	Model 1^b^	1	1.01 (0.86; 1.17)	0.90 (0.77; 1.05)	0.99 (0.85; 1.16)	1.01 (0.85; 1.18)	0.981
	Model 2^c^	1	1.00 (0.86; 1.18)	0.86 (0.74; 1.01)	0.95 (0.81; 1.12)	0.92 (0.78; 1.09)	0.262
	– healthy at baseline^d^ (*n*=10,795)	1	1.04 (0.85; 1.26)	0.82 (0.67; 1.00)	0.86 (0.70; 1.06)	0.98 (0.79; 1.20)	0.388
	– high risk^e^ (*n*=8507)	1	1.04 (0.88; 1.24)	0.88 (0.74; 1.05)	0.95 (0.80; 1.14)	1.00 (0.83; 1.20)	0.702
	– at least 3 days diary (*n*=12,815)	1	1.02 (0.87; 1.19)	0.87 (0.74; 1.02)	0.94 (0.80; 1.11)	0.93 (0.79; 1.10)	0.254
Postmenopausal Women (*n*=8176)							
CVD incidence (3306)	Person-Years	18648	18517	18397	18323	18724	
	Incidents	632	644	702	683	645	
	Model 1^b^	1	0.91 (0.82; 1.02)	0.96 (0.86; 1.07)	1.03 (0.92; 1.15)	1.02 (0.91; 1.14)	0.266
	Model 2^c^	1	0.89 (0.80; 0.99)	0.92 (0.83; 1.03)	0.99 (0.89; 1.11)	0.94 (0.84; 1.05)	0.793
	– healthy at baseline^d^ (*n*=5891)	1	0.94 (0.82; 1.09)	0.87 (0.75; 1.01)	0.93 (0.80; 1.08)	1.00 (0.87; 1.16)	0.976
	– high risk^e^ (*n*=5998)	1	0.90 (0.80; 1.02)	0.94 (0.84; 1.06)	1.00 (0.89; 1.13)	0.95 (0.84; 1.08)	0.901
	– at least 3 days diary (*n*=7839)	1	0.89 (0.80; 1.00)	0.92 (0.83; 1.03)	0.99 (0.88; 1.11)	0.95 (0.84; 1.06)	0.818
IHD incidence (1157)	Person-Years	20737	20796	20782	20578	21028	
	Incidents	218	226	250	259	204	
	Model 1^b^	1	0.94 (0.78; 1.13)	1.00 (0.84; 1.20)	1.15 (0.96; 1.38)	0.97 (0.80; 1.17)	0.620
	Model 2^c^	1	0.90 (0.75; 1.09)	0.96 (0.80; 1.16)	1.05 (0.87; 1.26)	0.84 (0.69; 1.02)	0.292
	– healthy at baseline^d^ (*n*=5891)	1	1.05 (0.80; 1.38)	0.92 (0.70; 1.21)	1.10 (0.84; 1.44)	0.94 (0.71; 1.24)	0.764
	– high risk^e^ (*n*=5998)	1	0.92 (0.75; 1.12)	0.97 (0.79; 1.18)	1.06 (0.87; 1.29)	0.88 (0.72; 1.09)	0.551
	– at least 3 days diary (*n*=7839)	1	0.93 (0.76; 1.12)	0.97 (0.80; 1.17)	1.05 (0.87; 1.27)	0.85 (0.70; 1.04)	0.311
Stroke incidence (5 5 0)	Person-Years	21517	21509	21576	21538	21685	
	Incidents	110	119	115	107	99	
	Model 1^b^	1	0.90 (0.69; 1.17)	0.81 (0.62; 1.05)	0.84 (0.64; 1.10)	0.89 (0.67; 1.16)	0.316
	Model 2^c^	1	0.89 (0.69; 1.16)	0.78 (0.60; 1.02)	0.79 (0.60; 1.04)	0.78 (0.59; 1.03)	0.054
	– healthy at baseline^d^ (*n*=5891)	1	0.92 (0.63; 1.33)	0.88 (0.61; 1.27)	0.87 (0.60; 1.26)	0.88 (0.60; 1.30)	0.494
	– high risk^e^ (*n*=5998)	1	0.95 (0.71; 1.25)	0.79 (0.59; 1.05)	0.80 (0.60; 1.08)	0.83 (0.61; 1.11)	0.117
	– at least 3 days diary (*n*=7839)	1	0.93 (0.71; 1.22)	0.81 (0.62; 1.07)	0.80 (0.61; 1.06)	0.83 (0.62; 1.10)	0.109
CVD mortality (7 7 6)	Person-Years	20488	20356	20554	20393	20753	
	Incidents	156	157	156	167	140	
	Model 1^b^	1	0.94 (0.72; 1.22)	0.77 (0.58; 1.01)	0.92 (0.70; 1.20)	0.89 (0.67; 1.18)	0.407
	Model 2^c^	1	0.93 (0.72; 1.22)	0.73 (0.56; 0.97)	0.85 (0.65; 1.12)	0.80 (0.60; 1.06)	0.089
	– healthy at baseline^d^ (*n*=5891)	1	1.03 (0.70; 1.52)	0.73 (0.48; 1.09)	0.74 (0.48; 1.12)	0.92 (0.61; 1.39)	0.326
	– high risk^e^ (*n*=5998)	1	0.99 (0.74; 1.31)	0.73 (0.54; 0.98)	0.89 (0.66; 1.19)	0.87 (0.64; 1.17)	0.248
	– at least 3 days diary (*n*=7839)	1	1.03 (0.78; 1.36)	0.77 (0.58; 1.02)	0.88 (0.66; 1.17)	0.86 (0.64; 1.16)	0.182
All cause mortality (2329)	Person-Years	20488	20356	20554	20393	20753	
	Incidents	444	484	477	492	432	
	Model 1^b^	1	0.97 (0.83; 1.14)	0.85 (0.72; 1.00)	0.96 (0.82; 1.13)	0.97 (0.82; 1.14)	0.670
	Model 2^c^	1	0.97 (0.83; 1.14)	0.83 (0.70; 0.98)	0.93 (0.79; 1.10)	0.89 (0.76; 1.06)	0.168
	– healthy at baseline^d^ (*n*=5891)	1	1.05 (0.85; 1.28)	0.74 (0.60; 0.92)	0.85 (0.69; 1.05)	0.91 (0.73; 1.13)	0.134
	– high risk^e^ (*n*=5998)	1	0.99 (0.83; 1.18)	0.86 (0.72; 1.03)	0.95 (0.79; 1.13)	0.95 (0.79; 1.142)	0.502
	– at least 3 days diary (*n*=7839)	1	1.01 (0.86; 1.19)	0.86 (0.73; 1.02)	0.94 (0.79; 1.11)	0.93 (0.78; 1.10)	0.263

a Missing values were assumed to be missing at random and were imputed using multiple imputation (*n*=5) with univariate regression models.

b Adjusted for BMI and energy intake.

c adjusted for BMI, energy intake, systolic blood pressure, plasma cholesterol, plasma vitamin C, intake of fiber, potassium, sodium, fat, saturated fat, and alcohol, and the following categorical variables–physical activity, smoking status, education, self-reported history of stroke, myocardial infarction and diabetes at baseline, family history of myocardial infarction, use of antihypertensive and lipid lowering drugs; additionally in women use of hormone replacement therapy.

d Includes only participants without self-reported history of stroke, myocardial infarct, diabetes mellitus, family history of myocardial infarct, and no use of antihypertensive and lipid-lowering drugs.

e Includes only participants with a 10-year CVD risk 20% higher than normal based on data from the Framingham Heart Study (estimate based on Framingham risk score using age, sex, BMI, and blood pressure [36].

## Footnotes

Supplementary data associated with this article can be found in the online version at doi:10.1016/j.freeradbiomed.2015.03.005.
