# Supplementary material for: Associations between flavan-3-ol intake and CVD risk in the Norfolk cohort of the European Prospective Investigation into Cancer (EPIC-Norfolk)
Source: Free Radic Biol Med. 2015 Jul;84:1–10. doi: 10.1016/j.freeradbiomed.2015.03.005 (PMC4503814; doi:10.1016/j.freeradbiomed.2015.03.005)
Supplement: Supplementary file 1 — Supplementary Data [file mmc1.doc]

**Supplemental Table 1:** Summary of missing data (absolute numbers and per-mille). For categorical data, an additional value of “missing” was created and assigned to missing observations. For continuous variables (cholesterol and plasma vitamin C), missing values were assumed to be missing at random and were imputed using multiple imputation (n=5) with univariate regression models.

|  | Men (n=11,273) | Women (n=13,634) |
| --- | --- | --- |
| Categorical variables |  |  |
| Physical activity | 1 (0.1‰) | — |
| Smoking status | 77 (6.8‰) | 133 (9.8‰) |
| Marital status | 56 (5.0‰) | 85 (6.2‰) |
| Education | 8 (0.7‰) | 9 (0.7‰) |
| Social class | 158 (14.0‰) | 301 (22.1‰) |
| Self-reported incidence of: | |  |
| Stroke | 10 (0.9‰) | 5 (0.4‰) |
| Myocardial infarct | 14 (1.2‰) | 6 (0.4‰) |
| Diabetes mellitus | 8 (0.7‰) | 8 (0.6‰) |
| Family history of myocardial infarct | 12 (1.1‰) | 11 (0.8‰) |
| Hormone replacement therapy | — | 15 (1.1‰) |
| Menopausal status | — | 15 (1.1‰) |
| Continuous variables |  |  |
| Vitamin C | 1280 (113.6‰) | 1753 (128.6‰) |
| Cholesterol | 663 (58.8‰) | 1014 (74.4‰) |

**Supplemental Table 2:** Baseline characteristics (mean and SD) of the study population in EPIC Norfolk by quintiles of flavan-3-ol intake. P value for one-way ANOVA or χ2 test.

|  | Men (n=11,252) | | | | | |
| --- | --- | --- | --- | --- | --- | --- |
|  | Q1 | Q2 | Q3 | Q4 | Q5 | p |
| n | 2251 | 2250 | 2251 | 2250 | 2250 |  |
| Flavan-3-ol intake [mg/d]  (Median and range) | 45  (0 – 114) | 160  (114 – 197) | 233  (197 – 268) | 309  (268 – 360) | 438  (360 – 3248) |  |
| Age [years] | 56.6 (9.0) | 59.7 (9.3) | 60.6 (9.4) | 60.0 (9.2) | 58.9 (9.1) | <0.0001 |
| BMI [kg/m^2^] | 26.9 (3.5) | 26.6 (3.3) | 26.5 (3.1) | 26.4 (3.2) | 26.3 (3.3) | <0.0001 |
| Systolic BP [mmHg] | 136 (18) | 138 (18) | 138 (18) | 138 (18) | 137 (17) | 0.0090 |
| Diastolic BP [mmHg] | 85 (11) | 84 (11) | 85 (11) | 84 (11) | 84 (11) | 0.6935 |
| Plasma cholesterol^†^ [mmol/L] | 6.1 (1.1) | 6.1 (1.1) | 6.1 (1.1) | 6.0 (1.1) | 5.9 (1.1) | <0.0001 |
| Plasma Vitamin C [µmol/L] | 48 (20) | 48 (19) | 48 (18) | 47 (18) | 45 (20) | <0.0001 |
| CVD Risk^‡^ (Framingham) | 21% (14%) | 24% (15%) | 25% (15%) | 24% (14%) | 24% (14%) | 0.0001 |
| Dietary intake | | | | | | |
| Energy [kJ/d] | 9280 (2050) | 9201 (1990) | 9286 (1977) | 9473 (1975) | 9775 (2061) | <0.0001 |
| Fibre [g/d] | 15 (6) | 16 (6) | 16 (6) | 17 (6) | 17 (6) | <0.0001 |
| Sodium [mg/d] | 3081 (873) | 3093 (822) | 3136 (808) | 3215 (797) | 3346 (877) | <0.0001 |
| Potassium [mg/d] | 3364 (809) | 3335 (749) | 3380 (731) | 3484 (735) | 3641 (785) | <0.0001 |
| Fat [g/d] | 84.7 (25.2) | 83.3 (24.0) | 84.6 (24.6) | 86.6 (24.5) | 89.3 (26.2) | <0.0001 |
| Saturated fat [g/d] | 32.2 (11.5) | 31.4 (10.6) | 31.9 (10.8) | 32.8 (10.9) | 33.8 (12.0) | <0.0001 |
| Alcohol [g/d] | 20.1 (24.1) | 20.1 (23.1) | 17.1 (21.1) | 14.1 (18.1) | 13.1 (19.1) | <0.0001 |
| Flavan-3-ol intake (geometric mean and SD) | | | | | | |
| Total flavan-3-ol [mg/d] | 198.4 (2.7) | 701.6 (1.2) | 1030.7 (1.1) | 1358.3 (1.1) | 2008.2 (1.2) | <0.0001 |
| Flavan-3-ol monomer [mg/d] | 38.3 (2.6) | 155.9 (1.2) | 231.7 (1.1) | 309.2 (1.1) | 463.7 (1.2) | <0.0001 |
| Epicatechin/Catechin [mg/d] | 20.9 (2.0) | 37.5 (1.3) | 47.0 (1.3) | 57.3 (1.2) | 76.8 (1.3) | <0.0001 |
| Gallated compounds [mg/d] | 6.5 (13.1) | 115.2 (1.3) | 182.9 (1.1) | 250.5 (1.1) | 385.6 (1.3) | <0.0001 |
| Epicatechin [mg/d] | 11.2 (2.1) | 20.9 (1.3) | 26.9 (1.3) | 33.2 (1.2) | 44.9 (1.3) | <0.0001 |
| Epicatechin-gallate [mg/d] | 0.6 (82.0) | 28.0 (1.6) | 44.2 (1.1) | 60.2 (1.1) | 92.4 (1.3) | <0.0001 |
| Epigallocatechin [mg/d] | 2.4 (12.1) | 37.3 (1.4) | 59.9 (1.2) | 82.2 (1.1) | 126.4 (1.3) | <0.0001 |
| Epigallocatechin-gallate [mg/d] | 0.8 (68.8) | 41.9 (2.0) | 68.5 (1.5) | 95.1 (1.1) | 147.0 (1.3) | <0.0001 |
| Catechin [mg/d] | 8.4 (3.2) | 16.0 (1.5) | 19.6 (1.3) | 23.6 (1.3) | 31.4 (1.3) | <0.0001 |
| Catechin-gallate [mg/d] | 0.0 (15.4) | 0.0 (14.7) | 0.0 (10.0) | 0.0 (9.7) | 0.0 (7.2) | 0.0005 |
| Gallocatechin [mg/d] | 0.1 (120.8) | 5.7 (2.0) | 9.2 (1.4) | 12.7 (1.1) | 19.5 (1.3) | <0.0001 |
| Proanthocyanidins [DP ≥ 2] [mg/d] | 83.2 (4.4) | 119.2 (1.8) | 133.2 (1.8) | 146.2 (1.7) | 168.8 (1.6) | <0.0001 |
| Theaflavins [mg/d] | 0.0 (10419.1) | 358.9 (4.6) | 630.9 (1.9) | 880.9 (1.1) | 1341.7 (1.4) | <0.0001 |
| Cocoa-Flavanols [mg/d] | 84.8 (2.3) | 127.5 (1.5) | 149.9 (1.5) | 172.4 (1.4) | 214.5 (1.4) | <0.0001 |
| Composition of dietary flavan-3-ols (contribution to total flavan-3-ols by individual compounds/classes) | | | | | | |
| Epicatechin/Catechin | 13% (11%) | 6% (3%) | 5% (1%) | 4% (1%) | 4% (1%) | <0.0001 |
| Gallated compounds | 8% (7%) | 17% (3%) | 18% (2%) | 19% (2%) | 19% (3%) | <0.0001 |
| Proanthocyanidins [DP ≥ 2] | 54% (28%) | 20% (11%) | 15% (8%) | 12% (6%) | 9% (5%) | <0.0001 |
| Theaflavins | 25% (25%) | 58% (10%) | 63% (7%) | 65% (5%) | 67% (6%) | <0.0001 |
| Smoking status |  |  |  |  |  |  |
| Current Smoker | 307 (14%) | 211 (9%) | 203 (9%) | 237 (11%) | 384 (17%) | <0.001 |
| Former Smoker | 1122 (50%) | 1259 (56%) | 1269 (56%) | 1225 (54%) | 1235 (55%) |  |
| Never Smoker | 807 (36%) | 764 (34%) | 765 (34%) | 770 (34%) | 618 (27%) |  |
| Physical Activity |  |  |  |  |  |  |
| Inactive | 680 (30%) | 654 (29%) | 718 (32%) | 742 (33%) | 669 (30%) | <0.001 |
| Moderately inactive | 592 (26%) | 626 (28%) | 565 (25%) | 499 (22%) | 496 (22%) |  |
| Moderately active | 529 (24%) | 531 (24%) | 525 (23%) | 511 (23%) | 513 (23%) |  |
| Active | 449 (20%) | 440 (20%) | 442 (20%) | 499 (22%) | 572 (25%) |  |
| Marital status |  |  |  |  |  |  |
| Single | 104 (5%) | 73 (3%) | 91 (4%) | 101 (4%) | 81 (4%) | <0.001 |
| Married | 1924 (85%) | 1968 (87%) | 1971 (88%) | 1975 (88%) | 1967 (87%) |  |
| Widowed | 59 (3%) | 79 (4%) | 64 (3%) | 85 (4%) | 62 (3%) |  |
| Separated | 27 (1%) | 22 (1%) | 22 (1%) | 12 (1%) | 23 (1%) |  |
| Divorced | 123 (5%) | 95 (4%) | 86 (4%) | 73 (3%) | 110 (5%) |  |
| Education level |  |  |  |  |  |  |
| None | 486 (22%) | 589 (26%) | 714 (32%) | 764 (34%) | 850 (38%) | <0.001 |
| O-Level | 232 (10%) | 227 (10%) | 178 (8%) | 169 (8%) | 166 (7%) |  |
| A-Level | 1078 (48%) | 1042 (46%) | 1014 (45%) | 1031 (46%) | 973 (43%) |  |
| Degree | 453 (20%) | 390 (17%) | 343 (15%) | 286 (13%) | 260 (12%) |  |
| Social class |  |  |  |  |  |  |
| Uncoded | 7 (0%) | 6 (0%) | 7 (0%) | 4 (0%) | 8 (0%) | <0.001 |
| Professional | 213 (9%) | 201 (9%) | 167 (7%) | 146 (6%) | 126 (6%) |  |
| Managerial | 1013 (45%) | 924 (41%) | 843 (37%) | 783 (35%) | 681 (30%) |  |
| Skilled - non-manual | 265 (12%) | 311 (14%) | 298 (13%) | 268 (12%) | 241 (11%) |  |
| Skilled - manual | 449 (20%) | 471 (21%) | 563 (25%) | 606 (27%) | 695 (31%) |  |
| Semiskilled | 225 (10%) | 250 (11%) | 281 (12%) | 334 (15%) | 379 (17%) |  |
| Non-skilled | 45 (2%) | 60 (3%) | 59 (3%) | 78 (3%) | 88 (4%) |  |
| Family history of MI | 793 (35%) | 763 (34%) | 796 (35%) | 830 (37%) | 808 (36%) | 0.567 |
| Self-reported history of |  |  |  |  |  |  |
| Stroke | 27 (1%) | 39 (2%) | 41 (2%) | 44 (2%) | 42 (2%) | 0.670 |
| MI | 98 (4%) | 132 (6%) | 120 (5%) | 127 (6%) | 118 (5%) | 0.390 |
| DM | 76 (3%) | 74 (3%) | 69 (3%) | 63 (3%) | 73 (3%) | 0.198 |
| Antihypertensive drug use | 351 (16%) | 437 (19%) | 448 (20%) | 481 (21%) | 391 (17%) | <0.001 |
| Lipid-lowering drug use | 39 (2%) | 38 (2%) | 33 (1%) | 29 (1%) | 29 (1%) | 0.600 |

^†^ To convert mmol/L into mg/dL, divide value by 0.0259; ^‡^ Estimate based Framingham risk score using age, sex, BMI and blood pressure**^(^**[**^1^**](#_ENREF_1)**^)^**

|  | Women (n=13,633) | | | | | |
| --- | --- | --- | --- | --- | --- | --- |
|  | Q1 | Q2 | Q3 | Q4 | Q5 | p |
| n | 2727 | 2727 | 2727 | 2727 | 2726 |  |
| Flavan-3-ol monomer [mg/d]  (Median and range) | 36  (0 – 102) | 150  (102 – 185) | 217  (185 – 248) | 283  (248 – 327) | 401  (327 – 2712) |  |
| Age [years] | 56.2 (8.9) | 59.3 (9.4) | 60.1 (9.4) | 59.4 (9.3) | 57.2 (9.0) | <0.0001 |
| BMI [kg/m^2^] | 26.6 (4.8) | 26.1 (4.3) | 26.2 (4.2) | 26.0 (4.1) | 26.2 (4.3) | <0.0001 |
| Systolic BP [mmHg] | 131 (18) | 134 (19) | 136 (19) | 135 (19) | 134 (19) | <0.0001 |
| Diastolic BP [mmHg] | 80 (11) | 81 (11) | 81 (11) | 81 (11) | 81 (11) | <0.0001 |
| Plasma cholesterol^†^ [mmol/L] | 6.3 (1.2) | 6.4 (1.2) | 6.3 (1.2) | 6.4 (1.2) | 6.2 (1.2) | <0.0001 |
| Plasma Vitamin C [µmol/L] | 59 (20) | 60 (20) | 59 (19) | 59 (20) | 57 (21) | <0.0001 |
| CVD Risk^‡^ (Framingham) | 15% (9%) | 16% (9%) | 16% (9%) | 16% (9%) | 15% (9%) | <0.0001 |
| Dietary intake |  |  |  |  |  |  |
| Energy [kJ/d] | 6941 (1544) | 6997 (1543) | 7090 (1476) | 7227 (1498) | 7363 (1542) | <0.0001 |
| Fibre [g/d] | 14 (5) | 14 (5) | 14 (5) | 15 (5) | 15 (5) | <0.0001 |
| Sodium [mg/d] | 2313 (659) | 2351 (621) | 2383 (607) | 2452 (613) | 2514 (663) | <0.0001 |
| Potassium [mg/d] | 2894 (695) | 2867 (647) | 2944 (625) | 3029 (643) | 3201 (707) | <0.0001 |
| Fat [g/d] | 62.6 (20.0) | 63.6 (19.6) | 64.0 (18.8) | 65.4 (19.3) | 66.4 (19.9) | <0.0001 |
| Saturated fat [g/d] | 23.7 (8.8) | 24.3 (8.9) | 24.4 (8.5) | 24.8 (8.7) | 25.1 (9.0) | <0.0001 |
| Alcohol [g/d] | 9.1 (13.1) | 9.1 (12.1) | 8.1 (12.1) | 7.1 (12.1) | 6.1 (11.1) | <0.0001 |
| Flavan-3-ol intake (geometric mean and SD) | | | | | | |
| Total flavan-3-ol [mg/d] | 179.3 (2.5) | 659.7 (1.2) | 966.4 (1.1) | 1254.1 (1.1) | 1828.6 (1.3) | <0.0001 |
| Flavan-3-ol monomer [mg/d] | 32.5 (2.5) | 145.8 (1.2) | 215.9 (1.1) | 284.0 (1.1) | 422.5 (1.3) | <0.0001 |
| Epicatechin/Catechin [mg/d] | 18.1 (1.9) | 33.1 (1.3) | 42.8 (1.2) | 51.8 (1.2) | 69.7 (1.3) | <0.0001 |
| Gallated compounds [mg/d] | 5.5 (12.9) | 110.6 (1.3) | 171.9 (1.1) | 231.2 (1.1) | 351.7 (1.3) | <0.0001 |
| Epicatechin [mg/d] | 10.2 (2.0) | 19.0 (1.6) | 25.0 (1.3) | 30.5 (1.2) | 41.1 (1.3) | <0.0001 |
| Epicatechin-gallate [mg/d] | 0.8 (56.6) | 28.0 (1.5) | 42.3 (1.2) | 56.4 (1.1) | 85.0 (1.3) | <0.0001 |
| Epigallocatechin [mg/d] | 1.9 (10.0) | 34.8 (1.4) | 55.9 (1.2) | 75.4 (1.1) | 114.7 (1.3) | <0.0001 |
| Epigallocatechin-gallate [mg/d] | 0.7 (45.7) | 38.3 (2.2) | 64.2 (1.2) | 87.2 (1.2) | 133.8 (1.3) | <0.0001 |
| Catechin [mg/d] | 6.9 (3.2) | 13.6 (1.4) | 17.5 (1.3) | 21.1 (1.2) | 28.4 (1.3) | <0.0001 |
| Catechin-gallate [mg/d] | 0.0 (67.9) | 0.0 (66.8) | 0.0 (46.8) | 0.0 (43.6) | 0.0 (36.5) | <0.0001 |
| Gallocatechin [mg/d] | 0.0 (142.4) | 5.1 (2.5) | 8.6 (1.2) | 11.5 (1.4) | 17.6 (1.3) | <0.0001 |
| Proanthocyanidins [DP ≥ 2] [mg/d] | 82.9 (4.7) | 114.4 (2.2) | 131.1 (1.7) | 143.6 (1.6) | 164.0 (1.6) | <0.0001 |
| Theaflavins [mg/d] | 0.0 (10621.1) | 314.0 (6.8) | 590.4 (1.8) | 800.6 (1.5) | 1208.0 (1.4) | <0.0001 |
| Cocoa-Flavanols [mg/d] | 79.3 (2.2) | 117.9 (1.6) | 141.3 (1.4) | 161.5 (1.4) | 200.4 (1.4) | <0.0001 |
| Composition of dietary flavan-3-ols | | | | | | |
| Epicatechin/Catechin | 11% (9%) | 5% (1%) | 4% (1%) | 4% (1%) | 4% (1%) | <0.0001 |
| Gallated compounds | 10% (8%) | 17% (3%) | 18% (2%) | 19% (2%) | 19% (3%) | <0.0001 |
| Proanthocyanidins [DP ≥ 2] | 51% (28%) | 19% (10%) | 15% (7%) | 12% (6%) | 10% (5%) | <0.0001 |
| Theaflavins | 29% (26%) | 59% (9%) | 63% (6%) | 65% (6%) | 67% (5%) | <0.0001 |
| Smoking status |  |  |  |  |  |  |
| Current Smoker | 383 (14%) | 237 (9%) | 218 (8%) | 264 (10%) | 407 (15%) | <0.001 |
| Former Smoker | 898 (33%) | 903 (33%) | 915 (34%) | 853 (31%) | 784 (29%) |  |
| Never Smoker | 1416 (52%) | 1558 (57%) | 1566 (57%) | 1586 (58%) | 1513 (56%) |  |
| Physical Activity |  |  |  |  |  |  |
| Inactive | 769 (28%) | 853 (31%) | 876 (32%) | 851 (31%) | 777 (29%) | 0.017 |
| Moderately inactive | 872 (32%) | 879 (32%) | 880 (32%) | 889 (33%) | 867 (32%) |  |
| Moderately active | 636 (23%) | 596 (22%) | 584 (21%) | 583 (21%) | 640 (23%) |  |
| Active | 450 (17%) | 399 (15%) | 387 (14%) | 404 (15%) | 442 (16%) |  |
| Marital status |  |  |  |  |  |  |
| Single | 119 (4%) | 114 (4%) | 100 (4%) | 87 (3%) | 117 (4%) | <0.001 |
| Married | 2041 (75%) | 2063 (76%) | 2060 (76%) | 2100 (77%) | 2079 (76%) |  |
| Widowed | 266 (10%) | 326 (12%) | 352 (13%) | 342 (13%) | 259 (10%) |  |
| Separated | 34 (1%) | 23 (1%) | 27 (1%) | 27 (1%) | 36 (1%) |  |
| Divorced | 251 (9%) | 176 (6%) | 171 (6%) | 157 (6%) | 222 (8%) |  |
| Education level |  |  |  |  |  |  |
| None | 943 (35%) | 1114 (41%) | 1226 (45%) | 1248 (46%) | 1214 (45%) | <0.001 |
| O-Level | 341 (13%) | 324 (12%) | 280 (10%) | 323 (12%) | 303 (11%) |  |
| A-Level | 1074 (39%) | 984 (36%) | 951 (35%) | 894 (33%) | 936 (34%) |  |
| Degree | 366 (13%) | 303 (11%) | 268 (10%) | 262 (10%) | 271 (10%) |  |
| Social class |  |  |  |  |  |  |
| Uncoded | 10 (0%) | 9 (0%) | 18 (1%) | 6 (0%) | 4 (0%) | <0.001 |
| Professional | 189 (7%) | 212 (8%) | 152 (6%) | 158 (6%) | 147 (5%) |  |
| Managerial | 1018 (37%) | 971 (36%) | 929 (34%) | 914 (34%) | 843 (31%) |  |
| Skilled - non-manual | 516 (19%) | 575 (21%) | 565 (21%) | 529 (19%) | 468 (17%) |  |
| Skilled - manual | 533 (20%) | 508 (19%) | 563 (21%) | 564 (21%) | 637 (23%) |  |
| Semiskilled | 315 (12%) | 298 (11%) | 356 (13%) | 372 (14%) | 433 (16%) |  |
| Non-skilled | 97 (4%) | 92 (3%) | 87 (3%) | 117 (4%) | 128 (5%) |  |
| Family history of MI | 1006 (37%) | 984 (36%) | 1027 (38%) | 1008 (37%) | 1040 (38%) | 0.916 |
| Self-reported history of |  |  |  |  |  |  |
| Stroke | 25 (1%) | 18 (1%) | 29 (1%) | 30 (1%) | 35 (1%) | 0.153 |
| MI | 31 (1%) | 36 (1%) | 39 (1%) | 36 (1%) | 34 (1%) | 0.912 |
| DM | 32 (1%) | 52 (2%) | 41 (2%) | 37 (1%) | 50 (2%) | 0.177 |
| Antihypertensive drug use | 418 (15%) | 553 (20%) | 557 (20%) | 562 (21%) | 457 (17%) | <0.001 |
| Lipid-lowering drug use | 39 (1%) | 43 (2%) | 44 (2%) | 33 (1%) | 43 (2%) | 0.719 |
| Menopausal status |  |  |  |  |  |  |
| Pre-menopausal | 583 (21%) | 401 (15%) | 364 (13%) | 396 (15%) | 531 (19%) | <0.001 |
| Peri-menopausal | 762 (28%) | 593 (22%) | 556 (20%) | 569 (21%) | 688 (25%) |  |
| Post-menopausal | 1377 (51%) | 1731 (63%) | 1803 (66%) | 1760 (65%) | 1505 (55%) |  |
| Hormone replacement therapy |  |  |  |  |  |  |
| Current | 642 (24%) | 590 (22%) | 517 (19%) | 495 (18%) | 511 (19%) | <0.001 |
| Former | 333 (12%) | 282 (10%) | 278 (10%) | 325 (12%) | 333 (12%) |  |
| Never | 1747 (64%) | 1853 (68%) | 1928 (71%) | 1905 (70%) | 1880 (69%) |  |

^†^ To convert mmol/L into mg/dL, divide value by 0.0259; ^‡^ Estimate based Framingham risk score using age, sex, BMI and blood pressure**^(^**[**^1^**](#_ENREF_1)**^)^**

|  | Post-menopausal women (n=8176) | | | | | |
| --- | --- | --- | --- | --- | --- | --- |
|  | Q1 | Q2 | Q3 | Q4 | Q5 | p |
| n | 1636 | 1635 | 1635 | 1635 | 1635 |  |
| Flavan-3-ol monomer [mg/d]  (Median and range) | 54  (0 – 119) | 159  (119 –191) | 219  (191 – 248) | 279  (248 – 320) | 384  (320 – 2712) |  |
| Age [years] | 63.2 (6.9) | 64.8 (6.6) | 65.3 (6.5) | 64.7 (6.4) | 63.5 (6.5) | <0.0001 |
| BMI [kg/m^2^] | 27.0 (4.8) | 26.5 (4.1) | 26.4 (4.1) | 26.3 (4.0) | 26.5 (4.2) | <0.0001 |
| Systolic BP [mmHg] | 137 (19) | 139 (19) | 140 (19) | 139 (19) | 139 (19) | <0.0001 |
| Diastolic BP [mmHg] | 82 (11) | 83 (11) | 83 (11) | 83 (11) | 83 (11) | 0.1912 |
| Plasma cholesterol^†^ [mmol/L] | 6.6 (1.2) | 6.7 (1.2) | 6.6 (1.2) | 6.7 (1.3) | 6.6 (1.1) | 0.0816 |
| Plasma Vitamin C [µmol/L] | 59 (21) | 59 (21) | 58 (20) | 58 (20) | 57 (21) | 0.0527 |
| CVD Risk^‡^ (Framingham) | 15% (9%) | 16% (9%) | 16% (9%) | 16% (9%) | 15% (9%) | 0.0001 |
| Dietary intake | | | | | | |
| Energy [kJ/d] | 6750 (1514) | 6872 (1464) | 6939 (1438) | 7063 (1491) | 7175 (1474) | <0.0001 |
| Fibre [g/d] | 13 (5) | 14 (5) | 14 (5) | 14 (5) | 15 (5) | <0.0001 |
| Sodium [mg/d] | 2267 (647) | 2327 (601) | 2359 (611) | 2404 (583) | 2474 (630) | <0.0001 |
| Potassium [mg/d] | 2822 (678) | 2818 (626) | 2872 (616) | 2952 (630) | 3105 (655) | <0.0001 |
| Fat [g/d] | 60.9 (19.2) | 62.6 (18.9) | 62.9 (18.3) | 63.7 (19.0) | 64.8 (19.0) | <0.0001 |
| Saturated fat [g/d] | 23.4 (8.8) | 24.0 (8.7) | 24.2 (8.4) | 24.4 (8.8) | 24.8 (8.8) | 0.0003 |
| Alcohol [g/d] | 8.1 (12.1) | 8.1 (11.1) | 7.1 (10.1) | 6.1 (11.1) | 5.1 (9.1) | <0.0001 |
| Flavan-3-ol intake (geometric mean and SD) | | | | | | |
| Total flavan-3-ol [mg/d] | 224.7 (2.5) | 708.4 (1.2) | 978.8 (1.1) | 1236.6 (1.1) | 1764.1 (1.2) | <0.0001 |
| Flavan-3-ol monomer [mg/d] | 42.3 (2.5) | 156.5 (1.1) | 218.8 (1.1) | 280.7 (1.1) | 406.1 (1.2) | <0.0001 |
| Epicatechin/Catechin [mg/d] | 19.6 (1.8) | 34.1 (1.3) | 42.4 (1.2) | 50.6 (1.2) | 67.1 (1.2) | <0.0001 |
| Gallated compounds [mg/d] | 10.2 (11.7) | 120.9 (1.2) | 175.5 (1.1) | 229.2 (1.1) | 338.1 (1.3) | <0.0001 |
| Epicatechin [mg/d] | 11.1 (2.2) | 19.9 (1.3) | 25.0 (1.2) | 30.0 (1.2) | 39.8 (1.3) | <0.0001 |
| Epicatechin-gallate [mg/d] | 1.8 (36.5) | 30.1 (1.3) | 43.0 (1.1) | 55.8 (1.1) | 81.6 (1.2) | <0.0001 |
| Epigallocatechin [mg/d] | 3.5 (9.4) | 38.8 (1.3) | 57.2 (1.1) | 74.7 (1.1) | 110.5 (1.2) | <0.0001 |
| Epigallocatechin-gallate [mg/d] | 1.7 (33.1) | 43.9 (1.5) | 65.8 (1.2) | 86.4 (1.2) | 128.6 (1.3) | <0.0001 |
| Catechin [mg/d] | 7.5 (2.7) | 13.9 (1.3) | 17.1 (1.3) | 20.4 (1.2) | 27.1 (1.2) | <0.0001 |
| Catechin-gallate [mg/d] | 0.0 (71.9) | 0.0 (48.9) | 0.0 (36.3) | 0.0 (34.2) | 0.0 (27.7) | <0.0001 |
| Gallocatechin [mg/d] | 0.1 (114.5) | 5.9 (1.5) | 8.8 (1.3) | 11.4 (1.5) | 17.0 (1.3) | <0.0001 |
| Proanthocyanidins [DP ≥ 2] [mg/d] | 87.5 (4.1) | 116.5 (1.8) | 128.3 (1.7) | 139.5 (1.6) | 159.7 (1.6) | <0.0001 |
| Theaflavins [mg/d] | 0.1 (8919.5) | 380.9 (3.8) | 605.0 (1.7) | 787.4 (1.7) | 1169.5 (1.4) | <0.0001 |
| Cocoa-Flavanols [mg/d] | 83.7 (2.1) | 119.6 (1.5) | 138.7 (1.4) | 157.1 (1.4) | 193.9 (1.4) | <0.0001 |
| Composition of dietary flavan-3-ols | | | | | | |
| Epicatechin/Catechin | 11% (9%) | 5% (1%) | 4% (1%) | 4% (1%) | 4% (1%) | <0.0001 |
| Gallated compounds | 10% (8%) | 17% (3%) | 18% (2%) | 19% (2%) | 19% (3%) | <0.0001 |
| Proanthocyanidins [DP ≥ 2] | 51% (28%) | 19% (10%) | 15% (7%) | 12% (6%) | 10% (5%) | <0.0001 |
| Theaflavins | 29% (26%) | 59% (9%) | 63% (6%) | 65% (6%) | 67% (5%) | <0.0001 |
| Smoking status |  |  |  |  |  |  |
| Current Smoker | 197 (12%) | 129 (8%) | 110 (7%) | 140 (9%) | 205 (13%) | <0.001 |
| Former Smoker | 573 (35%) | 559 (34%) | 578 (35%) | 532 (33%) | 472 (29%) |  |
| Never Smoker | 849 (52%) | 925 (57%) | 926 (57%) | 948 (58%) | 940 (57%) |  |
| Physical Activity |  |  |  |  |  |  |
| Inactive | 586 (36%) | 632 (39%) | 615 (38%) | 619 (38%) | 577 (35%) | 0.283 |
| Moderately inactive | 518 (32%) | 512 (31%) | 523 (32%) | 540 (33%) | 502 (31%) |  |
| Moderately active | 321 (20%) | 300 (18%) | 309 (19%) | 291 (18%) | 348 (21%) |  |
| Active | 211 (13%) | 191 (12%) | 188 (12%) | 185 (11%) | 208 (13%) |  |
| Marital status |  |  |  |  |  |  |
| Single | 76 (5%) | 70 (4%) | 65 (4%) | 53 (3%) | 77 (5%) | <0.001 |
| Married | 1124 (69%) | 1182 (72%) | 1188 (73%) | 1193 (73%) | 1192 (73%) |  |
| Widowed | 272 (17%) | 283 (17%) | 289 (18%) | 295 (18%) | 247 (15%) |  |
| Separated | 15 (1%) | 7 (0%) | 12 (1%) | 11 (1%) | 14 (1%) |  |
| Divorced | 141 (9%) | 79 (5%) | 77 (5%) | 75 (5%) | 100 (6%) |  |
| Education level |  |  |  |  |  |  |
| None | 672 (41%) | 808 (49%) | 867 (53%) | 893 (55%) | 848 (52%) | <0.001 |
| O-Level | 175 (11%) | 147 (9%) | 136 (8%) | 153 (9%) | 163 (10%) |  |
| A-Level | 595 (36%) | 529 (32%) | 512 (31%) | 470 (29%) | 521 (32%) |  |
| Degree | 194 (12%) | 151 (9%) | 120 (7%) | 119 (7%) | 103 (6%) |  |
| Social class |  |  |  |  |  |  |
| Uncoded | 11 (1%) | 8 (0%) | 12 (1%) | 4 (0%) | 4 (0%) | <0.001 |
| Professional | 123 (8%) | 125 (8%) | 71 (4%) | 75 (5%) | 77 (5%) |  |
| Managerial | 583 (36%) | 514 (31%) | 529 (32%) | 498 (30%) | 471 (29%) |  |
| Skilled - non-manual | 363 (22%) | 397 (24%) | 359 (22%) | 355 (22%) | 314 (19%) |  |
| Skilled - manual | 268 (16%) | 307 (19%) | 322 (20%) | 337 (21%) | 364 (22%) |  |
| Semiskilled | 180 (11%) | 179 (11%) | 233 (14%) | 233 (14%) | 275 (17%) |  |
| Non-skilled | 58 (4%) | 60 (4%) | 64 (4%) | 79 (5%) | 78 (5%) |  |
| Family history of MI | 646 (39%) | 652 (40%) | 633 (39%) | 618 (38%) | 662 (40%) | 0.207 |
| Self-reported history of |  |  |  |  |  |  |
| Stroke | 29 (2%) | 15 (1%) | 24 (1%) | 25 (2%) | 32 (2%) | 0.364 |
| MI | 33 (2%) | 30 (2%) | 33 (2%) | 28 (2%) | 31 (2%) | 0.849 |
| DM | 28 (2%) | 41 (3%) | 31 (2%) | 26 (2%) | 40 (2%) | 0.272 |
| Antihypertensive drug use | 373 (23%) | 421 (26%) | 434 (27%) | 430 (26%) | 395 (24%) | 0.066 |
| Lipid-lowering drug use | 35 (2%) | 40 (2%) | 34 (2%) | 29 (2%) | 39 (2%) | 0.694 |
| Hormone replacement therapy |  |  |  |  |  |  |
| Current | 320 (20%) | 260 (16%) | 222 (14%) | 218 (13%) | 219 (13%) | <0.001 |
| Former | 237 (14%) | 181 (11%) | 171 (10%) | 215 (13%) | 216 (13%) |  |
| Never | 1079 (66%) | 1194 (73%) | 1242 (76%) | 1202 (74%) | 1200 (73%) |  |

^†^ To convert mmol/L into mg/dL, divide value by 0.0259; ^‡^ Estimate based Framingham risk score using age, sex, BMI and blood pressure**^(^**[**^1^**](#_ENREF_1)**^)^**

Supplemental Table 3: Association between flavan-3-ol intake and systolic and diastolic blood pressure (log-transformed) at baseline. Regression coefficient (β) and 95% confidence interval^†^. Statistically significant associations (p<0.05) are highlighted in bold.

|  |  | Q2 | Q3 | Q4 | Q5 | p_Trend_ |
| --- | --- | --- | --- | --- | --- | --- |
| Systolic blood pressure [log(mmHg)] | | | | | | |
| Men | | | | | | |
| Model 1^‡^ | 11,252 | -0.003 (-0.010; 0.004) | -0.002 (-0.009; 0.005) | 0.001 (-0.006; 0.007) | -0.002 (-0.009; 0.005) | 0.866 |
| Model 2^§^ | 11,252 | -0.002 (-0.009; 0.005) | 0.000 (-0.007; 0.006) | 0.002 (-0.005; 0.009) | 0.000 (-0.007; 0.007) | 0.751 |
| - healthy at baseline^#^ | 8636 | -0.002 (-0.009; 0.006) | -0.006 (-0.013; 0.002) | 0.000 (-0.008; 0.008) | -0.003 (-0.011; 0.004) | 0.541 |
| - high risk^¶^ | 8010 | -0.001 (-0.008; 0.006) | -0.003 (-0.010; 0.005) | -0.002 (-0.009; 0.006) | -0.004 (-0.012; 0.003) | 0.251 |
| - at least three days diary | 10,343 | -0.005 (-0.012; 0.003) | 0.001 (-0.006; 0.008) | 0.000 (-0.008; 0.007) | -0.001 (-0.008; 0.007) | 0.792 |
| Women | | | | | | |
| Model 1^‡^ | 13,633 | 0.007 (0.001; 0.014) | 0.012 (0.005; 0.018) | 0.011 (0.004; 0.018) | 0.013 (0.006; 0.019) | <0.001 |
| Model 2^§^ | 13,633 | 0.006 (-0.001; 0.012) | 0.010 (0.003; 0.016) | 0.008 (0.002; 0.015) | 0.010 (0.003; 0.016) | 0.002 |
| - healthy at baseline^#^ | 10,795 | 0.009 (0.002; 0.017) | 0.012 (0.005; 0.019) | 0.009 (0.002; 0.016) | 0.013 (0.005; 0.020) | 0.002 |
| - high risk^¶^ | 8507 | 0.000 (-0.007; 0.007) | 0.006 (-0.001; 0.013) | 0.005 (-0.002; 0.012) | 0.003 (-0.004; 0.010) | 0.18 |
| - at least three days diary | 12,815 | 0.006 (-0.001; 0.012) | 0.010 (0.003; 0.016) | 0.008 (0.001; 0.015) | 0.010 (0.003; 0.017) | 0.004 |
| Post-menopausal women | | | | | | |
| Model 1^‡^ | 8176 | 0.006 (-0.003; 0.014) | 0.013 (0.004; 0.022) | 0.008 (-0.001; 0.017) | 0.016 (0.007; 0.025) | <0.001 |
| Model 2^§^ | 8176 | 0.003 (-0.006; 0.012) | 0.010 (0.001; 0.019) | 0.004 (-0.004; 0.013) | 0.012 (0.003; 0.021) | 0.008 |
| - healthy at baseline^#^ | 5891 | 0.006 (-0.004; 0.016) | 0.011 (0.001; 0.021) | 0.003 (-0.008; 0.013) | 0.014 (0.004; 0.025) | 0.018 |
| - high risk^¶^ | 5998 | -0.002 (-0.011; 0.006) | 0.005 (-0.003; 0.014) | 0.004 (-0.004; 0.013) | 0.004 (-0.005; 0.012) | 0.177 |
| - at least three days diary | 7839 | 0.004 (-0.005; 0.013) | 0.010 (0.001; 0.019) | 0.004 (-0.005; 0.013) | 0.012 (0.003; 0.021) | 0.012 |
| Diastolic blood pressure [log(mmHg)] | | | | | | |
| Men | | | | | | |
| Model 1^‡^ | 11,252 | -0.003 (-0.011; 0.004) | 0.000 (-0.008; 0.007) | -0.003 (-0.010; 0.005) | -0.002 (-0.010; 0.005) | 0.608 |
| Model 2^§^ | 11,252 | -0.002 (-0.010; 0.005) | 0.001 (-0.007; 0.008) | -0.001 (-0.008; 0.007) | 0.000 (-0.007; 0.008) | 0.823 |
| - healthy at baseline^#^ | 8636 | 0.000 (-0.008; 0.008) | -0.005 (-0.013; 0.003) | -0.002 (-0.010; 0.006) | -0.004 (-0.012; 0.005) | 0.335 |
| - high risk^¶^ | 8010 | -0.002 (-0.010; 0.006) | 0.000 (-0.008; 0.008) | -0.004 (-0.012; 0.004) | -0.002 (-0.010; 0.006) | 0.567 |
| - at least three days diary | 10,343 | -0.006 (-0.014; 0.002) | 0.001 (-0.007; 0.009) | -0.004 (-0.012; 0.004) | -0.001 (-0.009; 0.007) | 0.999 |
| Women | | | | | | |
| Model 1^‡^ | 13,633 | 0.005 (-0.002; 0.012) | 0.007 (0.000; 0.014) | 0.008 (0.002; 0.015) | 0.012 (0.005; 0.019) | 0.001 |
| Model 2^§^ | 13,633 | 0.004 (-0.003; 0.011) | 0.006 (-0.001; 0.013) | 0.007 (0.000; 0.014) | 0.010 (0.003; 0.017) | 0.004 |
| - healthy at baseline^#^ | 10,795 | 0.009 (0.001; 0.016) | 0.008 (0.001; 0.016) | 0.007 (-0.001; 0.014) | 0.014 (0.007; 0.022) | 0.001 |
| - high risk^¶^ | 8507 | 0.001 (-0.006; 0.009) | 0.004 (-0.004; 0.011) | 0.006 (-0.002; 0.013) | 0.003 (-0.004; 0.011) | 0.216 |
| - at least three days diary | 12,815 | 0.004 (-0.003; 0.011) | 0.006 (-0.001; 0.013) | 0.007 (0.000; 0.014) | 0.010 (0.003; 0.017) | 0.005 |
| Post-menopausal women | | | | | | |
| Model 1^‡^ | 8176 | 0.002 (-0.007; 0.011) | 0.006 (-0.003; 0.015) | 0.004 (-0.005; 0.013) | 0.011 (0.002; 0.020) | 0.016 |
| Model 2^§^ | 8176 | 0.000 (-0.009; 0.009) | 0.004 (-0.005; 0.013) | 0.001 (-0.008; 0.010) | 0.008 (-0.001; 0.017) | 0.082 |
| - healthy at baseline^#^ | 5891 | 0.002 (-0.008; 0.012) | 0.005 (-0.006; 0.015) | -0.001 (-0.012; 0.009) | 0.011 (0.001; 0.022) | 0.079 |
| - high risk^¶^ | 5998 | -0.004 (-0.013; 0.005) | 0.001 (-0.008; 0.010) | 0.003 (-0.006; 0.012) | -0.001 (-0.011; 0.008) | 0.835 |
| - at least three days diary | 7839 | 0.001 (-0.008; 0.010) | 0.004 (-0.005; 0.013) | 0.002 (-0.007; 0.011) | 0.008 (-0.001; 0.017) | 0.105 |

^†^ missing values were assumed to be missing at random and were imputed using multiple imputation (n=5) with univariate regression models; ^‡^adjusted for age, BMI and energy intake; ^§^ adjusted for age, BMI, energy intake, plasma vitamin C, intake of fibre, fat, saturated fat and alcohol and the following categorical variables: physical activity, smoking status, marital status, education, use of anti-hypertensive drugs and self-reported history of stroke, myocardial infarction and diabetes at baseline, family history of myocardial infarction and – for women – menopausal status and hormone replacement therapy; ^#^includes only participants without self-reported history of stroke, myocardial infarct, diabetes mellitus, family history of myocardial infarct and no use of antihypertensive and lipid-lowering drugs. ^¶^includes only participants with a 10-year CVD risk 20% higher than normal based on data from the Framingham Heart Study (estimate based Framingham risk score using age, sex, BMI and blood pressure **(**[**35**](#_ENREF_35)**)**

Supplemental table 4: Observational studies of associations between flavan-3-ol intake and cardio-vascular disease risks.

| Study^†^ | Compounds | n | Sex | Endpoint | | Diet assessment | Intake^‡^ [mg/d] | | RR (Q1 vs Q5)^❡^ |
| --- | --- | --- | --- | --- | --- | --- | --- | --- | --- |
| IWHa | Flavan-3-ol monomers | 32,857 | F^§^ | CHD | Mortality | FFQ | 4^m^ | 75^m^ | 0.85 (0.67; 1.07) |
|  | Catechin + Epicatechin |  |  |  |  |  | 3^m^ | 23^m^ | 0.76 (0.58; 1.03) |
| ZE | Flavan-3-ol monomers | 806 | M | IHD | Mortality | Diet History | 25^m^ | 124^m^ | 0.49 (0.27; 0.88) |
|  |  |  | M | MI | Incidence |  |  |  | 0.70 (0.39; 1.26) |
|  |  |  | M | Stroke | Mortality |  |  |  | 0.81 (0.36; 1.83) |
|  |  |  | M | Stroke | Incidence |  |  |  | 0.92 (0.51; 1.68) |
| IWHb | Flavan-3-ol monomers + Thearubigins | 34,489 | F^§^ | Total | Mortality | FFQ | 4 | 182 | 0.98 (0.91; 1.06) |
|  |  |  |  | Stroke | Mortality |  |  |  | 0.95 (0.71; 1.28) |
|  |  |  |  | CHD | Mortality |  |  |  | 1.02 (0.86; 1.21) |
| NHS I | Flavan-3-ol monomers | 46,672 | F | Hypertension | Incidence | FFQ | 9^m^ | 176^m^ | 0.98 (0.93; 1.03) |
| NHS II |  | 87,242 | F |  |  |  | 10^m^ | 196^m^ | 1.03 (0.97; 1.09) |
| HPFS |  | 23,043 | M |  |  |  | 12^m^ | 150^m^ | 1.12 (1.02; 1.22) |
| NHS | Flavan-3-ol monomers | 69622 | F | Stroke | Incidence | FFQ | <12 | >76 | 0.87 (0.72; 1.06) |
| CPS II | Flavan-3-ol monomers | 38,180 | M | CVD | Mortality | FFQ | 7 | 64^m^ | 0.87 (0.74; 1.02) |
|  |  | 60,289 | F^#^ |  |  |  |  |  | 0.79 (0.66; 0.94) |

^†^IWHa: Iowa Women’s Health a; ^‡^intake in bottom and top quantile, median (except for ^m^: mean, ^p25/p75^: inter-quartile range); ^❡^Relative risk and 95% confidence interval, bottom *vs* top quantile, multivariable model; ^§^post-menopausal only; ^#^peri- and post-menopausal only.

Supplemental Table 5: Summary of sub-acute and chronic (duration 14d and more) dietary intervention studies investigating the effect of flavan-3-ol intake on systolic blood pressure. Only studies included in the recent Cochrane review^(^[^2^](#_ENREF_2)^)^, except for * which were published later

| Study | n | Intervention food | Intervention food [mg/d]^†^ | | | Blinded | Duration [d] | ΔSBP  (mean ± SEM) |
| --- | --- | --- | --- | --- | --- | --- | --- | --- |
|  |  |  | Cocoa Flavanols | PA | Epicatechin |  |  |  |
| Taubert, 2003^(^[^3^](#_ENREF_3)^)^ | 13 | Chocolate | 227e | 143e | 66e | No | 14 | -5.1 ± 0.7 |
| Murphy, 2003^(^[^4^](#_ENREF_4)^)^ | 28 | Other | 234 | 187e | 28e | Yes | 28 | -1.0 ± 4.0 |
| Engler, 2004^(^[^5^](#_ENREF_5)^)^ | 21 | Chocolate | 259 | 213 | 46 | Yes | 14 | 1.8 ± 4.4 |
| Fraga, 2005 | 28 | Chocolate | 168 | 126 | 30 | No | 14 | -4.0 ± 1.6 |
| Grassi, 2005a^(^[^6^](#_ENREF_6)^)^ | 15 | Chocolate | 227e | 143e | 66 | No | 15 | -6.5 ± 1.5 |
| Grassi, 2005b^(^[^6^](#_ENREF_6)^)^ | 20 | Chocolate | 227e | 143e | 66 | No | 15 | -11.3 ± 1.0 |
| Taubert, 2007^(^[^7^](#_ENREF_7)^)^ | 44 | Chocolate | 18e | 11 | 5 | No | 126 | -2.8 ± 2.3 |
| Crews, 2007 | 90 | Chocolate | 755 | 603e | 75e | Yes | 42 | -0.5 ± 2.6 |
| Grassi, 2008^(^[^8^](#_ENREF_8)^)^ | 19 | Chocolate | 382e | 241e | 111 | No | 15 | -3.7 ± 0.7 |
| Muniyappa, 2008^(^[^9^](#_ENREF_9)^)^ | 20 | Drink | 902 | 676 | 174 | Yes | 14 | -1.0 ± 1.6 |
| Davison, 2008a^(^[^10^](#_ENREF_10)^)^ | 21 | Drink | 902 | 676 | 174 | Yes | 84 | -6.1 ± 3.5 |
| Davison, 2008b^(^[^10^](#_ENREF_10)^)^ | 26 | Drink | 902 | 676 | 174 | Yes | 84 | 1.6 ± 4.5 |
| Al-Faris, 2008 | 60 | Chocolate | 12e | 8e | 4 | No | 15 | -7.1 ± 2.2 |
| Shiina, 2009 | 39 | Chocolate | 550 | 346e | 160e | No | 14 | 2.9 ± 6.6 |
| Ried, 2009 | 21 | Chocolate | 85e | 54e | 25e | No | 56 | 3.0 ± 2.7 |
| Monagas, 2009^(^[^11^](#_ENREF_11)^)^ | 25 | Other | 472e | 426 | 46 | No | 28 | 0.6 ± 3.8 |
| Bogaard, 2010^(^[^12^](#_ENREF_12)^)^ | 41 | Drink | 305 | 267 | 25 | Yes | 21 | 0.3 ± 1.5 |
| Heiss, 2010^(^[^13^](#_ENREF_13)^)^ | 16 | Drink | 375 | 310 | 59 | Yes | 30 | -5.0 ± 3.2 |
| Davison, 2010a^(^[^14^](#_ENREF_14)^)^ | 27 | Drink | 372 | 275 | 28 | Yes | 42 | -0.3 ± 2.9 |
| Davison, 2010b^(^[^14^](#_ENREF_14)^)^ | 28 | Drink | 712 | 530 | 43 | Yes | 42 | 0.9 ± 1.7 |
| Davison, 2010c^(^[^14^](#_ENREF_14)^)^ | 29 | Drink | 1052 | 785 | 58 | Yes | 42 | -4.4 ± 1.7 |
| Njijke, 2011 | 38 | Drink | 805 | 736 | 48 | Yes | 42 | 3.2 ± 1.7 |
| *Grassi, 2012a | 60 | Drink | 990 | 746 | 185 | Yes | 56 | -8.7 ± 0.5 |
| *Grassi, 2012b | 60 | Drink | 520 | 390 | 95 | Yes | 56 | -6.8 ± 0.5 |
| *Flammer, 2012 | 20 | Chocolate | 37e | 23e | 11 | Yes | 28 | -6.9 ± 3.6 |
| *Desideri, 2012a^(^[^15^](#_ENREF_15)^)^ | 60 | Drink | 520 | 390 | 95 | Yes | 56 | -5.5 ± 0.8 |
| *Desideri, 2012b^(^[^15^](#_ENREF_15)^)^ | 60 | Drink | 993 | 746 | 185 | Yes | 56 | -10.0 ± 1.2 |

^†^ Flavan-3-ol intake from compound specific analyses; “e” indicates that intake was estimated using food composition data.

# References

1. D'Agostino RB, Sr., Vasan RS, Pencina MJ *et al.* (2008) General cardiovascular risk profile for use in primary care: the Framingham Heart Study. *Circulation* **117**, 743-753.

2. Ried K, Sullivan T, Fakler P *et al.* (2012) Effect of cocoa on blood pressure. *Cochrane Database of Systematic Reviews*, 1-83.

3. Taubert D, Berkels R, Roesen R *et al.* (2003) Chocolate and blood pressure in elderly individuals with isolated systolic hypertension. *JAMA: The Journal of the American Medical Association* **290**, 1029-1030.

4. Murphy KJ, Chronopoulos AK, Singh I *et al.* (2003) Dietary flavanols and procyanidin oligomers from cocoa (Theobroma cacao) inhibit platelet function. *Am J Clin Nutr* **77**, 1466-1473.

5. Engler MB, Engler MM, Chen CY *et al.* (2004) Flavonoid-rich dark chocolate improves endothelial function and increases plasma epicatechin concentrations in healthy adults. *J Am Coll Nutr* **23**, 197-204.

6. Grassi D, Necozione S, Lippi C *et al.* (2005) Cocoa reduces blood pressure and insulin resistance and improves endothelium-dependent vasodilation in hypertensives. *Hypertension* **46**, 398-405.

7. Taubert D, Roesen R, Lehmann C *et al.* (2007) Effects of low habitual cocoa intake on blood pressure and bioactive nitric oxide: a randomized controlled trial. *JAMA: The Journal of the American Medical Association* **298**, 49-60.

8. Grassi D, Desideri G, Necozione S *et al.* (2008) Blood pressure is reduced and insulin sensitivity increased in glucose-intolerant, hypertensive subjects after 15 days of consuming high-polyphenol dark chocolate. *J Nutr* **138**, 1671-1676.

9. Muniyappa R, Hall G, Kolodziej TL *et al.* (2008) Cocoa consumption for 2 wk enhances insulin-mediated vasodilatation without improving blood pressure or insulin resistance in essential hypertension. *American Journal of Clinical Nutrition* **88**, 1685-1696.

10. Davison K, Coates AM, Buckley JD *et al.* (2008) Effect of cocoa flavanols and exercise on cardiometabolic risk factors in overweight and obese subjects. *International Journal of Obesity* **32**, 1289-1296.

11. Monagas M, Khan N, Andres-Lacueva C *et al.* (2009) Effect of cocoa powder on the modulation of inflammatory biomarkers in patients at high risk of cardiovascular disease. *American Journal of Clinical Nutrition* **90**, 1144-1150.

12. van den Bogaard B, Draijer R, Westerhof BE *et al.* (2010) Effects on Peripheral and Central Blood Pressure of Cocoa With Natural or High-Dose Theobromine: A Randomized, Double-Blind Crossover Trial. *Hypertension* **56**, 839-846.

13. Heiss C, Jahn S, Taylor M *et al.* (2010) Improvement of endothelial function with dietary flavanols is associated with mobilization of circulating angiogenic cells in patients with coronary artery disease. *J Am Coll Cardiol* **56**, 218-224.

14. Davison K, Berry NM, Misan G *et al.* (2010) Dose-related effects of flavanol-rich cocoa on blood pressure. *Journal of human hypertension* **24**, 568-576.

15. Desideri G, Kwik-Uribe C, Grassi D *et al.* (2012) Benefits in Cognitive Function, Blood Pressure, and Insulin Resistance Through Cocoa Flavanol Consumption in Elderly Subjects With Mild Cognitive Impairment: The Cocoa, Cognition, and Aging (CoCoA) Study. *Hypertension*.
